# Antigenicity, Immunogenicity and Protective Efficacy of Three Proteins Expressed in the Promastigote and Amastigote Stages of *Leishmania infantum* against Visceral Leishmaniasis

**DOI:** 10.1371/journal.pone.0137683

**Published:** 2015-09-14

**Authors:** Vivian Tamietti Martins, Miguel Angel Chávez-Fumagalli, Daniela Pagliara Lage, Mariana Costa Duarte, Esther Garde, Lourena Emanuele Costa, Viviane Gomes da Silva, Jamil Silvano Oliveira, Danielle Ferreira de Magalhães-Soares, Santuza Maria Ribeiro Teixeira, Ana Paula Fernandes, Manuel Soto, Carlos Alberto Pereira Tavares, Eduardo Antonio Ferraz Coelho

**Affiliations:** 1 Departamento de Bioquímica e Imunologia, Instituto de Ciências Biológicas, Universidade Federal de Minas Gerais, Belo Horizonte, Minas Gerais, Brazil; 2 Programa de Pós-Graduação em Ciências da Saúde: Infectologia e Medicina Tropical, Faculdade de Medicina, Universidade Federal de Minas Gerais, Belo Horizonte, Minas Gerais, Brazil; 3 Departamento de Patologia Clínica, COLTEC, Universidade Federal de Minas Gerais, Belo Horizonte, Minas Gerais, Brazil; 4 Centro de Biología Molecular Severo Ochoa, CSIC-UAM, Departamento de Biología Molecular, Universidad Autónoma de Madrid, Madrid, Spain; 5 Departamento de Medicina Veterinária Preventiva, Escola de Veterinária, Universidade Federal de Minas Gerais, Belo Horizonte, Minas Gerais, Brazil; 6 Departamento de Análises Clínicas e Toxicológicas, Faculdade de Farmácia, Universidade Federal de Minas Gerais, Belo Horizonte, Minas Gerais, Brazil; Philipps-University Marburg, GERMANY

## Abstract

In the present study, two *Leishmania infantum* hypothetical proteins present in the amastigote stage, LiHyp1 and LiHyp6, were combined with a promastigote protein, IgE-dependent histamine-releasing factor (HRF); to compose a polyproteins vaccine to be evaluated against *L*. *infantum* infection. Also, the antigenicity of the three proteins was analyzed, and their use for the serodiagnosis of canine visceral leishmaniasis (CVL) was evaluated. The LiHyp1, LiHyp6, and HRF DNA coding sequences were cloned in prokaryotic expression vectors and the recombinant proteins were purified. When employed in ELISA assays, all proteins were recognized by sera from visceral leishmaniasis (VL) dogs, and presented no cross-reactivity with either sera from dogs vaccinated with a Brazilian commercial vaccine, or sera of *Trypanosoma cruzi*-infected or *Ehrlichia canis*-infected animals. In addition, the antigens were not recognized by antibodies from non-infected animals living in endemic or non-endemic areas for leishmaniasis. The immunogenicity and protective efficacy of the three proteins administered in the presence of saponin, individually or in combination (composing a polyproteins vaccine), were evaluated in a VL murine model: BALB/c mice infected with *L*. *infantum*. Spleen cells from mice inoculated with the individual proteins or with the polyproteins vaccine plus saponin showed a protein-specific production of IFN-γ, IL-12, and GM-CSF after an *in vitro* stimulation, which was maintained after infection. These animals presented significant reductions in the parasite burden in different evaluated organs, when compared to mice inoculated with saline or saponin. The decrease in parasite burden was associated with an IL-12-dependent production of IFN-γ against parasite total extracts (produced mainly by CD4^+^ T cells), correlated to the induction of parasite proteins-driven NO production. Mice inoculated with the recombinant protein-based vaccines showed also high levels of parasite-specific IgG2a antibodies. The polyproteins vaccine administration induced a more pronounced Th1 response before and after challenge infection than individual vaccines, which was correlated to a higher control of parasite dissemination to internal organs.

## Introduction

Visceral leishmaniasis (VL) represents an important disease in the world, leading to nearly 50,000 deaths annually [1]. The primary choice for the treatment of disease is based on the parenteral administration of pentavalent antimonials; however, parasites’ increased resistance and side effects have been registered in the patients as important problems [2,3]. Other drugs, such as amphotericin B and its liposomal formulations, as well as paramomycin and miltefosine, have shown encouraging results; however, their use is commonly related to toxicity and/or high cost [4]. Therefore, the development of new strategies to prevent VL has become a priority [5].

Canine visceral leishmaniasis (CVL) caused by *Leishmania infantum* is a major global zoonosis. Upon infection, dogs can develop distinct clinical manifestations of the disease: asymptomatic, oligosymptomatic, or symptomatic stages [6,7,8]. Symptomatic CVL usually results in death, and the clinical manifestations are varied, ranging from cutaneous alterations to neurological disorders [7,9,10]. Infected dogs can also remain asymptomatic, and even be classified as false-negative in both clinical evaluations and serological trials performed [8]. This is an important problem, since infected dogs (even asymptomatic ones) are important domestic reservoirs of parasites, and can further contribute to transmission between sand flies and humans [11]. In this context, a precise and early diagnosis of CVL is of utmost importance [12].

As described in detail previously [13], in active VL, the cell-mediated immune response is absent and in the patients that are cured, the Th1 type response is increased, leading to long time immunity [14]. This provides a rationale that Th1 response play a major role in prevention and/or cure of VL. Therefore, proteins that stimulate the Th1 type arm of the immune response could be exploited as vaccine candidates against VL [15–21]. The induction of CD4^+^ Th1 cells response for parasite antigens is crucial in controlling infection. Cytokines like IFN-γ are able to induce the production of nitric oxide and other compounds by infected phagocityc cells, thereby assisting to control of the parasites´ multiplication [21,22]. On the contrary, IL-4, IL-10, IL-13, and TGF-β represent important disease promoting cytokines, leading in turn to the suppression of the Th1 response and contributing to the disease [23,24]. Concomitantly to the role of CD4^+^ T cells, the cytotoxic activity performed by CD8^+^ T cells also contributes to protection against VL. These cells were linked to act against re-infection, but studies have also showed that CD8^+^ T cells act also with an important role in controlling the primary infection, by increasing the Th1 response [20,25–27].

Protozoan parasites of the genus *Leishmania* have a dimorphic life cycle, consisting of extracellular promastigotes that multiply and develop within the alimentary tract of the sand fly vector, and intracellular amastigotes that multiply within the phagolysosomes of their host macrophages [6,27–29]. The most studies on *Leishmania spp*. vaccines have focused on promastigote antigens. On the other hand, as described in detail previously [30], amastigote antigens have been far less tested as vaccine candidates against VL. However, the amastigotes seem to be the more appropriate target for the immune responses elicited by a vaccine, since after a few hours of initial infection and during the active disease, only this parasite stage is present in the host tissues. In addition, in contrast to promastigotes, the amastigote forms reside inside host cells and are targets for CD8^+^ T cells, elements involved in the protective immunity against VL [31]. In this context, aiming to produce a vaccine composed by both amastigote and promastigote antigens; the present study evaluated three proteins: LiHyp1 (XP_001468941.1), LiHyp6 (XP_001467126.1) and the IgE-dependent histamine-releasing factor (HRF) (CAJ05086.1). The first two proteins were identified within the proteins extracted from amastigote-like cells by an immunoproteomic approach using sera from asymptomatic and symptomatic VL dogs [5]. The last one was characterized by the same procedure, but employing promastigote extracts [5].

The LiHyp1 protein (36.6 kDa) was recently shown to be protective against *L*. *infantum* [13]. In the present work, and in order to increase its prophylactic efficacy, this antigen was combined with other new characterized antigens for developing a new composition. The LiHyp6 gene is predicted to encode a protein with a theoretical molecular weight of 21.4 kDa, while the HRF gene is predicted to encode a protein with 19.4 kDa. Interestingly, mammals´ HRF protein, has been described as having a stimulatory activity on the immune system of the hosts by activating both T and B cells [32,33]. Therefore, the first purpose of this study is to analyze the antigenicity of the three recombinant proteins using canine patients. In addition, the immunogenicity and protective properties of the three proteins, administered individually or in combination, in the presence of an adjuvant able to induce cellular response; have been also studied.

## Material and Methods

### Ethics Statement

Experiments were performed in compliance with the National Guidelines of the Institutional Animal Care, and Committee on the Ethical Handling of Research Animals (CEUA) from the Federal University of Minas Gerais (UFMG) (Law number 11.794, 2008), with code number 043/2011. In addition, the owners of the domestic dogs (*Canis familiaris*) gave permission to collect blood samples from their animals.

### Canine sera

Dog blood samples (10 mL) were collected by venipuncture of jugular vein in tubes without anticoagulant, and were kept at 37°C by 15 min, when they were centrifuged at 3,000 × *g* for 15 min, and sera samples were separated and kept at −80°C, until use. The sample size of domestic animals was 86 domestic animals (*Canis familiaris*) and consisted of males (n = 44) and females (n = 42) of different breeds and ages. CVL-positive animals were selected on the basis of two serological tests (IFAT [IFAT- LVC Bio-Manguinhos kit] and ELISA [EIE-LVC Bio-Manguinhos kit], both from Biomanguinhos, Fiocruz, Brazil), for *Leishmania spp*. Dogs with an IFAT titre < 1/40 or ELISA reactivity below the cut-off value indicated by the manufacturer were considered to be seronegative. Animals with an IFAT titre > 1/40 and an ELISA value over the cut-off were considered to be seropositive. Thus, symptomatic VL dogs (n = 15) were those positive by IFAT and ELISA, and when submitted to PCR, also presented positive results for *L*. *infantum* kDNA; and presenting three or more of the following clinical symptoms: weight loss, alopecia, adenopathy, onychogryposis, hepatomegaly, conjunctivitis; and exfoliative dermatitis on the nose, tail, and ear tips. Asymptomatic VL dogs (n = 9) presented positive serological (IFAT and ELISA) and parasitological (PCR) results, but they do not present clinical signs or symptoms of leishmaniasis. Non-infected dogs were selected from an endemic (n = 15; Belo Horizonte, Minas Gerais, Brazil) or non-endemic (n = 15; Poços de Caldas, Minas Gerais, Brazil) area of leishmaniasis, but they presented negative serological (IFAT and ELISA) results, as well as present any clinical signs or symptoms of leishmaniasis. Non-infected animals immunized with the Leish-Tec^®^ vaccine (n = 12), which were isolated in kennels to prevent their contact with transmitting vectors of leishmaniasis; as well as dogs experimentally infected with *Trypanosoma cruzi* (n = 12) or *Ehrlichia canis* (n = 8), were used in the ELISA assays.

### Mice, parasite and antigen preparation

Female BALB/c mice (8 weeks age) were obtained from the breeding facilities of the Department of Biochemistry and Immunology, Institute of Biological Sciences, UFMG; and were maintained under specific pathogen-free conditions. Experiments were carried out using the *L*. *infantum* (MOM/BR/1970/BH46) strain. Parasites were grown at 24°C in Schneider's medium (Sigma, St. Louis, MO, USA), which was supplemented with 10% heat-inactivated fetal bovine serum (FBS, Sigma), 20 mM L-glutamine, 100 U/mL penicillin, and 50 μg/mL streptomycin, at pH 7.4. The soluble *L*. *infantum* antigenic extract (SLA) was prepared from 10^9^ stationary-phase promastigotes, like previously described [34]. Briefly, parasites were washed three times in 5 mL of cold sterile phosphate-buffered saline (PBS). After seven cycles of freezing (-196°C) and thawing (+37°C), the suspension was centrifuged at 8,000 x *g* for 20 min at 4°C; and supernatant containing SLA was collected in 500 μL aliquots, being stored at -80°C, until used. The protein concentration was estimated by the Bradford method [35].

### Cloning, expression, and purification of recombinant proteins

LiHyp1 was cloned, expressed, and the recombinant protein was purified as previously described [13]. The primers used to amplify the LiHyp6 and HRF genes from *L*. *infantum* genomic DNA were: 5´-TTTGCTAGCATGAGCTTCTTTGACTTCTCA-3’ (*forward*) and 5´-TTTAAGCTTTCATTGCAGAACTTTGAGTACA-3´ (*reverse*) for LiHyp6, and 5´-GGATCCATGAAGATCTTCAAGGATGTG-3´ (*forward*) and 5´- AAGCTTAGACGCGCTCGCCCTTCAG-3´ (*reverse*) for HRF proteins. Cut sites for *Bam*HI and *Hin*dIII (underlined) were included for cloning purposes in both cases. For PCR, genomic DNA from *L*. *infantum* was used. After amplification, DNA fragments were excised from gels, purified, and linked into pGEM-T easy vector systems (Promega, USA). Recombinant plasmids were used to transform *Escherichia coli* XL1-Blue (Phoneutria, Brazil) competent cells. Positive clones were tested by restriction analysis with *Eco*RI, and those presenting LiHyp6 or HRF genes were propagated, double-stranded sequenced, and used in the construction of the expression vector. DNA fragments obtained from *Nhe*I*/Eco*RI and *Bam*HI*/Hin*dIII digestion of pGEM-LiHyp6 or pGEM-HRF plasmids were ligated into the corresponding cut sites of the pET-28a-c and pQE30 plasmids, respectively (Qiagen, Hilden, Germany), and transformed into BL21AI and M15 *E*. *coli* strains for overexpression induced by adding 1 mM isopropyl-β-D-thiogalactopyranoside (IPTG) (Promega, Montreal, Canada). After induction, cells were ruptured by five cycles of ultrasound (15 sec each, 90 MHz) in a binding buffer (0.02 M phosphate buffer, pH 8.0, 0.5 M NaCl, 0.005 M imidazole, 8 M urea, and 0.001 M β-mercaptoethanol). The presence of the recombinant proteins in the supernatant extracts after centrifugation (13,000 × *g*, 20 min at 4°C) was analyzed by one dimensional SDS-PAGE. Both proteins proved to be soluble and had been purified under native conditions, following manufacturer’s instructions (Qiagen). Briefly, rLiHyp6 and rHFR proteins were transferred by gravity flow onto Ni-nitrilotriacetic-acid-agarose (Ni-NTA) columns. The recombinant proteins were successively washed (1 column vol.) in binding buffer and in wash buffer (0.02 M phosphate buffer pH 8.0, 0.5 M NaCl, 0.005 M imidazole, and 0.001 M β-mercaptoethanol), and eluted in elution buffer (0.02 M phosphate buffer pH 8.0, 0.5 M NaCl, 0.5 M imidazole, and 0.001 M β-mercaptoethanol). After elution, proteins were dialyzed against 1x phosphate saline buffer (PBS). The rLiHyp6 and rHRF proteins were concentrated in eppendorf Vacufuge vacuum concentrate, and further purified on a Superdex^TM^ 200 gel-filtration column (GE Healthcare Life Sciences, USA). After purification, the recombinant proteins were passed through a polymyxin-agarose column (Sigma) to remove any residual endotoxins content. The purity of the recombinant proteins was checked by an one dimensional SDS-PAGE.

### Validation of the purified proteins by Western-Blot assays

To verify the antigenicity of the purified proteins in the CVL; the individual rLiHyp1, rLiHyp6, and rHRF proteins (10 μg each) were submitted to a 12% SDS-PAGE and blotted onto a nitrocellulose membrane (0.2 μm pore size, Sigma, St. Louis, USA), when they were evaluated in reaction to sera samples of VL dogs, or sera from non-infected animals (negative control). The technical protocol was performed as previously described [13].

### ELISA experiments for the serodiagnosis of CVL

Previous titration curves were performed to determine the most appropriate antigen concentration and antibody dilution to be used. For the serodiagnosis of CVL, the rLiHyp1, rLiHyp6, rHRF, and SLA-specific IgG antibodies levels were evaluated using a canine serological panel. For this, microtiter immunoassay plates (Falcon) were coated with the individual recombinant proteins (1.5, 1.5, or 0.5 μg per well, for rLiHyp1, rLiHyp6, and rHRF, respectively), with their mixture (0.5 μg per well, of each protein), or SLA *L*. *infantum* (1.0 μg per well); all diluted in 100 μL of coating buffer (50 mM carbonate buffer pH 9.6), for 18 h at 4°C. After washing the plates three times with PBS-T (PBS plus Tween 20 0.05%), their free binding sites were blocked using 200 μL of PBS-T containing 2% casein, for 1 h at 37°C. After washed three times with PBS-T, plates were incubated with 100 μL of individual canine sera (1:200, diluted in PBS-T), for 1 h at 37°C. Plates were subsequently washed four times in PBS-T, and incubated with anti-dog IgG horseradish-peroxidase conjugated antibody (1:10,000, diluted in PBS-T; catalog A6792, Sigma Aldrich, USA), for 1 h at 37°C. After washing the plates four times with PBS-T, the reaction was developed by incubation with 100 μL per well of a solution composed by 2 μL H_2_O_2_, 2 mg orto-phenylenediamine, and 10 mL citrate-phosphate buffer at pH 5.0 for 30 min in the dark. The reaction was stopped by adding 25 μL 2 N H_2_SO_4_, and the optical density was read in an ELISA microplate spectrophotometer (Molecular Devices, Spectra Max Plus, Canada), at 492 nm.

### Vaccination regimens, challenge infection and determination of parasite burden

Mice (n = 8, per group) were vaccinated subcutaneously in their left hind footpad with 25 μg of each recombinant protein (rLiHyp1, rLiHyp6, or rHRF), or with their mixture (using 25 μg of each protein), all associated with 25 μg of saponin (*Quillaja saponaria* bark saponin, Sigma). Additional mice were immunized with saponin or received saline. Three doses of the vaccines were administered at two-week intervals into the animals. Four weeks after the third and last immunization, mice (n = 4 per group) were euthanized and sera samples and spleen were collected to analyze the immune response induced by vaccination. At the same time, the remaining animals of each group were infected subcutaneously in the right hind footpad with 10^7^ stationary-phase promastigotes of *L*. *infantum*. Ten weeks after infection, animals were euthanized and sera samples, spleen, liver, bone marrow (BM), and draining lymph nodes (dLN) were collected. Spleen and blood were used to evaluate the generated immune response. For the evaluation of the parasite burden, spleen, liver, BM and dLN were processed and used in a limiting-dilution technique, as previously described [36]. In this case, triplicates instead of duplicates were used in the parasitological analysis.

### Cytokine production before and after *L*. *infantum* infection

Splenocyte cultures were performed four weeks after the third and last immunization and before infection, as well as in the 10^th^ week after challenge, as described [34]. Briefly, single-cell suspensions from spleen tissue were plated in duplicate in 24-well plates (Nunc), at 10^6^ cells per mL. Cells were incubated in RPMI 1640 medium (negative control), which was supplemented with 10% FBS, 20 mM L-glutamine, 200 U/mL penicillin, and 100 μg/mL streptomycin, at pH 7.4; or separately stimulated with rLiHyp1, rLiHyp6 or rHRF proteins (20 μg mL^-1^, each); with their mixture (10 μg mL^-1^ of each protein), or with SLA *L*. *infantum* (25 μg mL^-1^), for 48 h at 37°C in 5% CO_2_. IFN-γ, IL-4, IL-10, IL-12, and GM-CSF levels were assessed in the supernatants by a sandwich ELISA provided in commercial kits (BD OptEIA TM set mouse IFN-γ, IL-4, IL-10, IL-12, and GM-CSF; all obtained from Pharmingen, San Diego, CA, USA); following manufacturer’s instructions. To evaluate the involvement of IL-12, and CD4^+^ and CD8^+^ T cells, spleen cells of mice vaccinated with rLiHyp1/saponin, rLiHyp6/saponin, rHRF/saponin or polyproteins/saponin and infected were *in vitro* stimulated with SLA *L*. *infantum* (25 μg mL^-1^), and incubated in the presence of 5 μg mL^-1^ of monoclonal antibodies (mAb) against mouse IL-12 (C17.8), CD4 (GK 1.5), or CD8 (53–6.7). Appropriate isotype-matched controls—rat IgG2a (R35-95) and rat IgG2b (95–1)–were employed in the assays. Antibodies (no azide/low endotoxin^TM^) were purchased from BD (Pharmingen).

### Humoral response and nitrite production

For the analysis of the humoral response induced after vaccination, the rLiHyp1, rLiHyp6, rHRF, and SLA-specific IgG1 and IgG2a isotypes levels were evaluated using the sera samples collected from the vaccinated animals, four weeks after the third and last immunization and before infection; as well as in the 10^th^ week after challenge, by an ELISA technique, as previously described [13]. The used concentration of the recombinant proteins were: 1.0, 1.0, and 0.5 μg per well for rLiHyp1, rLiHyp6, and rHRF proteins, respectively; 0.5 μg per well of each protein to their mixture, and 2.0 μg per well of SLA *L*. *infantum*. The sera samples were diluted at 1:200, and the anti-mouse IgG1 and IgG2a horseradish-peroxidase conjugated antibodies (Sigma-Aldrich, USA) were employed both in a 1:5,000 dilution. The nitrite production in the cultures supernatant was assessed by the Griess reaction [37]. Data were expressed as μM per 10^6^ cells.

### Statistical analysis

The results were entered into Microsoft Excel (version 10.0) spreadsheets and analyzed using GraphPad Prism^TM^ (version 6.0 for Windows). The mean optical density (OD) value was calculated by subtracting the mean blank OD from OD mean for each sample by using specific values obtained in the ELISA assays. The lower limit of positivity (cut-off) for SLA *L*. *infantum*, rLiHyp1, rLiHyp6, rHRF, and their mixture was established for optimal sensitivity and specificity using the Receiver Operator Curve (ROC curve). The accuracy was evaluated according to the area under the curve (AUC) relative to the ROC curve, 95% confidence interval (95%CI). The ROC curves were plotted with the values from serum samples from dogs presenting symptomatic and asymptomatic CVL as compared to those from the control groups (*T*. *cruzi*-infected, *E*. *canis*-infected, Leish-Tec-immunized, and non-infected from endemic and non-endemic area dogs), according to a sick/non-sick rating method, in which one inclusion criterion in each group was the positivity or negativity of PCR for *L*. *infantum* kDNA in blood samples. Statistical analysis of the data from vaccinated and/or infected mice was performed by one-way analysis of variance (ANOVA), using Tukey’s post-test for multiple comparisons between the groups. The mean ± standard deviation (SD) of each experimental group is shown. Differences were considered significant when *P* < 0.05. The vaccination experiments were repeated once, and presented similar results. Data showed in this study are representative of one of them.

## Results

### Serodiagnosis of CVL using the recombinant proteins

**Fig 1:**
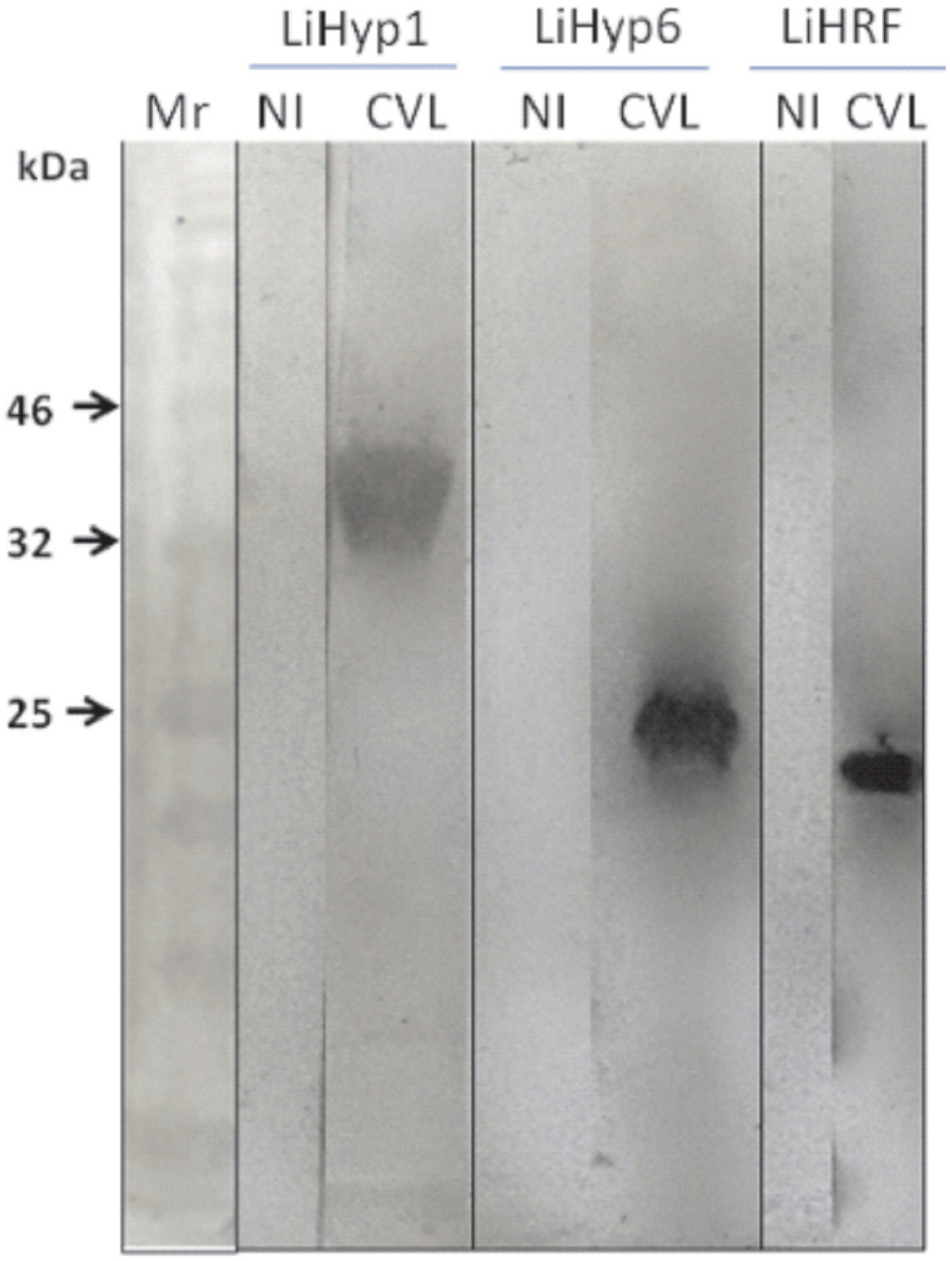
Immunoblotting experiments using the recombinant proteins and a canine serological panel. For immunoblotting experiments, the rLiHyp1 (36.6 kDa), rLiHyp6 (21.4 kDa), and rHRF (19.4 kDa) proteins (10 μg, each) were submitted to a 12% SDS-PAGE and blotted onto a nitrocellulose membrane, which were blocked with a PBS-T containing 5% BSA solution, and incubated with a pool of sera of asymptomatic and symptomatic VL dogs, or with a pool containing sera of non-infected dogs (1:200 and 1:100 diluted in PBS-T, respectively). Peroxidase conjugated anti-dog IgG (1:10,000) was used as a second antibody. The reactivities against the rLiHyp1, rLiHyp6 and rHRF proteins are shown. A low range protein ladder (Invitrogen^TM^, Life Technologies, USA) was used (Mr). The individual reactions of the rLiHyp1, rLiHyp6, and rHRF proteins with the pools of sera from non-infected or *L*. *infantum*-infected dogs (NI and CVL, respectively) are shown. Immunoblottings were derived from three independent experiments, and one representative preparation is showed in this study.

**Fig 2:**
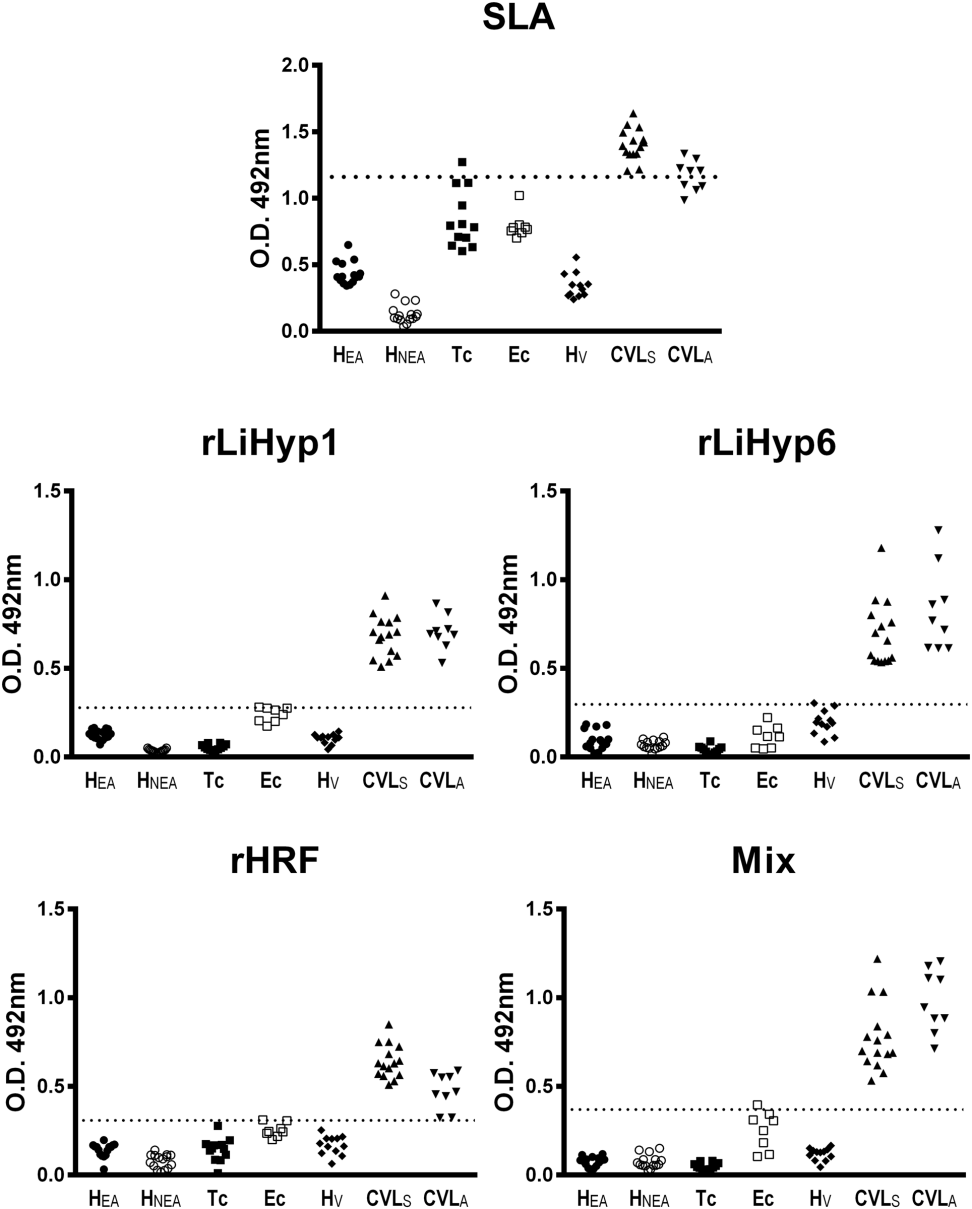
Evaluation of the ELISA reactivity using the recombinant proteins against different sera samples. ELISA experiments were performed using sera samples from *T*. *cruzi*-infected (n = 19), asymptomatic (n = 20) and symptomatic (n = 25) *L*. *infantum*-infected, and non-infected (n = 40) dogs. Reactions against SLA *L*. *infantum* (1.0 μg per well) (**A**), rLiHyp1 (1.5 μg per well) (**B**), rLiHyp6 (1.5 μg per well) (**C**), rHRF (0.5 μg per well) (**D**) and their mixture (0.5 μg per well of each protein) (**E**), were performed. The mean optical density (OD) value was calculated by subtracting the mean blank O.D. from O.D. mean for each sample, by using specific values obtained in the ELISA assays. The lower limit of positivity (cut-off) for rLiHyp1, rLiHyp6, rHRF, their mixture, and SLA *L*. *infantum* was established for optimal sensitivity and specificity using the ROC curves. Abbreviations: H_EA_: healthy from endemic area, H_NEA_: healthy from non-endemic area, Tc: *Trypanosoma cruzi*, Ec: *Ehrlichia canis*, H_V_: healthy and vaccinated, CVL_S_: symptomatic canine visceral leishmaniasis, CVL_A_: asymptomatic canine visceral leishmaniasis.

Initially, to analyze the antigenicity of the recombinant proteins, a Western-blot assay was performed. As it is shown (Fig 1), the rLiHyp1, rLiHyp6, and rHRF proteins were recognized by the sera from CVL-infected animals (in the right, in Fig 1). However, when sera from non-infected dogs were used, no significant reactivity was observed with the evaluated three recombinant proteins (in the left, in Fig 1). In this context, antigens were evaluated individually or in combination in ELISA experiments to determine their sensitivity and specificity for the serodiagnosis of CVL (Fig 2). The SLA *L*. *infantum* was used as a control and a comparative antigen. In the results, all CVL sera recognized the SLA antigen, but a significant cross-reactivity was observed when sera samples from *T*. *cruzi*-infected animals were evaluated (Fig 2A). On the contrary, the rLiHyp1 (Fig 2B), rLiHyp6 (Fig 2C), and rHRF (Fig 2D) proteins were recognized by all asymptomatic and symptomatic CVL sera, but no cross-reactivity with sera from *T*. *cruzi*-infected animals was observed. When the three proteins were used as a mixture, it was able to better distinguish between the CVL sera from the other sera groups (Fig 2E). To determine the diagnostic performance of the rLiHyp1, rLiHyp6 and rHRF proteins, as well as their mixture; ROC curves were constructed. The proteins presented sensitivity and specificity values of 100.0% and 98.4%, respectively; whereas using SLA *L*. *infantum*, these values were of 83.3% and 98.47%, respectively. It was concluded that the mixture of the three proteins presented a better performance than SLA for the serodiagnosis of CVL (Table 1).

**Table 1:** Diagnostic performance of recombinant proteins and their mixture using a serological panel. Samples from asymptomatic (n = 9) or symptomatic (n = 15) *L*. *infantum*-infected dogs, sera from animals with no clinical signs of VL, and negative parasitological and serological results to *Leishmania spp*. antigens, and living in an endemic (n = 15) or non-endemic (n = 15) area of leishmaniasis; as well as sera from animals experimentally infected by *Trypanosoma cruzi* (n = 12) or *Ehrlichia canis* (n = 8), and sera from dogs vaccinated with Leish-Tec (n = 12); were tested in the ELISA assays. ROC curves were used to determine sensitivity, specificity and AUC (area under the curve). 95%CI: Confiance Interval. LR: Likehood Ratio. SLA: Soluble *Leishmania infantum* antigenic extract.

Antigen	AUC	95%CI	Se	95%CI	Sp	95%CI	LR
SLA	0.99	0.9–1.0	83.3	62.6–95.3	98.4	91.3–100.0	51.7
rLiHyp1	1.0	1.0–1.0	100.0	85.8–100.0	98.4	91.3–100.0	62.0
rLiHyp6	1.0	1.0–1.0	100.0	85.8–100.0	98.4	91.3–100.0	62.0
rHRF	1.0	1.0–1.0	100.0	85.8–100.0	98.4	91.3–100.0	62.0
Mix	1.0	1.0–1.0	100.0	85.8–100.0	98.4	91.3–100.0	62.0

### Immunogenicity of the recombinant proteins in BALB/c mice

**Fig 3:**
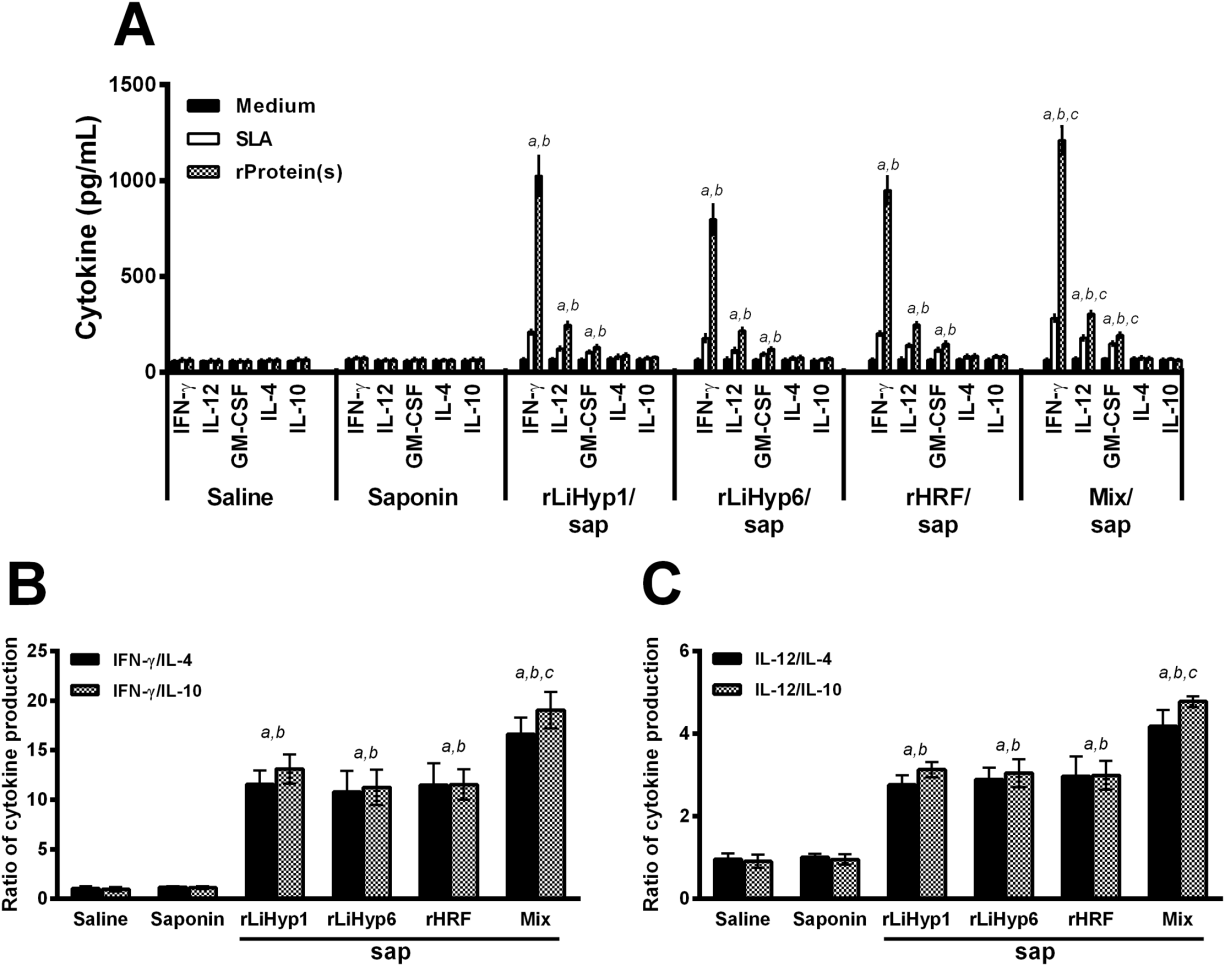
Cellular response induced in BALB/c mice by immunization using the recombinant proteins plus saponin. Mice (n = 8, per group) were vaccinated subcutaneously in their left hind footpad with 25 μg of each recombinant protein (rLiHyp1, rLiHyp6, or rHRF), or with their mixture (using 25 μg of each protein), all associated with 25 μg of saponin (*Quillaja saponaria* bark saponin, Sigma). Additional mice were immunized with saponin or received saline. Three doses were administered, at two-week intervals. Four weeks after the last immunization, mice (n = 4 per group) were euthanized and spleens were collected to evaluate the cellular response induced by vaccination. For this, single cell suspensions were incubated in RPMI 1640 medium (negative control), which was supplemented with 10% FBS, 20 mM L-glutamine, 200 U/mL penicillin, and 100 μg/mL streptomycin, at pH 7.4; or separately stimulated with rLiHyp1 (rLiHyp1/saponin group), rLiHyp6 (rLiHyp6/saponin group), rHRF (rHRF/saponin group) proteins (20 μg mL^-1^, each one); with their mixture (saline, saponin, and polyproteins/saponin groups; using 10 μg mL^-1^ of each protein), or SLA *L*. *infantum* (25 μg mL^-1^), for 48 h at 37°C in 5% CO_2_. IFN-γ, IL-12, GM-CSF, IL-4, and IL-10 levels were measured by ELISA in the culture supernatants (**A**). Bars represent the mean ± standard deviation (SD) of the groups. In addition, the ratios between IFN-γ/IL-4 and IFN-γ/IL-10 levels (**B**), and between IL-12/IL-4 and IL-12/IL-10 levels (**C**) were calculated and are shown. (*a*) indicates statistically significant difference in relation to the saline group (*P* < 0.001). (*b*) indicates statistically significant difference in relation to the saponin group (*P* < 0.001). (*c*) indicates statistically significant difference in relation to the rLiHyp1/saponin, rLiHyp6/saponin, and rHRF/saponin groups (*P* < 0.001).

**Fig 4:**
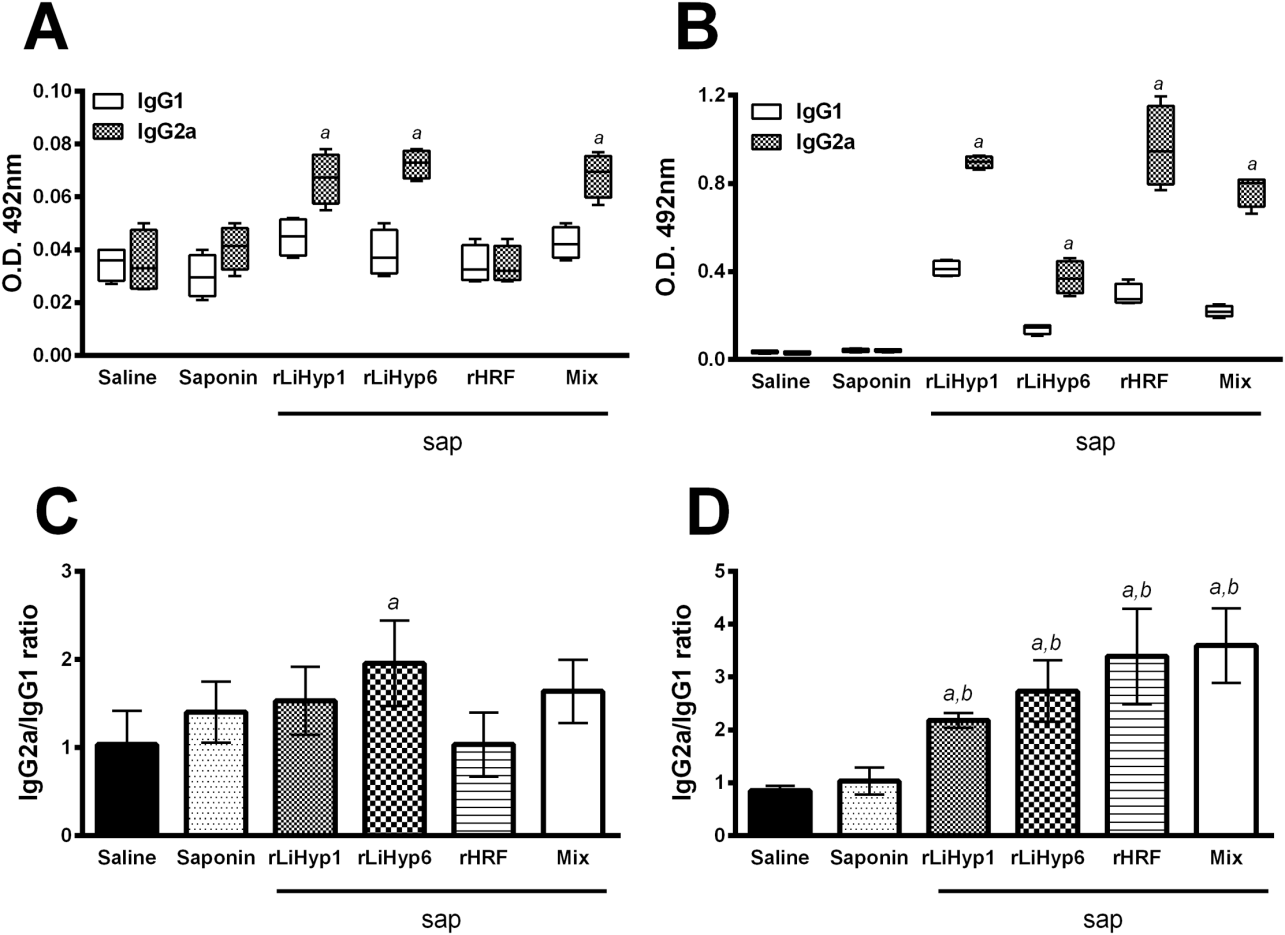
Humoral response generated in BALB/c mice by immunization with the recombinant proteins plus saponin. Mice (n = 8, per group) were vaccinated subcutaneously in their left hind footpad with 25 μg of each recombinant protein (rLiHyp1, rLiHyp6, or rHRF), or with their mixture (using 25 μg of each protein), all associated with 25 μg of saponin (*Quillaja saponaria* bark saponin, Sigma). Additional mice were immunized with saponin or received saline. Three doses were administered, at two-week intervals. Four weeks after the last immunization, mice (n = 4 per group) were euthanized and sera samples were collected to evaluate the humoral response induced by vaccination. The reactivity based on IgG1 and IgG2a isotypes represented by whisker (min to max) plots against SLA (**A**) and the respective recombinant proteins (**B**) are shown. In each group vaccinated with the recombinant protein, the respective antigen was used. In the saline and saponin groups, as well as in the polyproteins/saponin group, the protein mixture was used as antigen. Also, SLA was used as antigen in all groups. For the assays, 1.0, 1.0, and 0.5 μg per well of rLiHyp1, rLiHyp6, and rHRF proteins, respectively; 0.5 μg per well of each protein composing the antigenic mixture, and 2.0 μg per well of SLA *L*. *infantum*, were used in the ELISA assays. The sera samples were diluted at 1:200, and the anti-mouse IgG1 and IgG2a horseradish-peroxidase conjugated antibodies were employed in a 1:5,000 and 1:10,000 dilution, respectively. The ratios between the IgG2a/IgG1 levels against SLA (**C**) and the respective recombinant proteins (**D**) were calculated, and are also shown. Bars represent the mean ± standard deviation (SD) of the groups. (*a*) indicates statistically significant difference in relation to the saline group (*P* < 0.001). (*b*) indicates statistically significant difference in relation to the saponin group (*P* < 0.001).

The immunogenicity of rLiHyp1, rLiHyp6, and rHRF proteins administered with saponin individually or in a mixed formulation was evaluated in BALB/c mice (Fig 3). Cellular responses against the antigens were analyzed four weeks after receiving three vaccine doses, and just before infection. Following *in vitro* stimulation with recombinant proteins used in the immunizations, spleen cells from vaccinated mice with rLiHyp1, rLiHyp6, or rHRF plus saponin produced significantly higher levels of IFN-γ, IL-12, and GM-CSF than those secreted by spleen cells of the animals that received saline or saponin (stimulated with the protein mixture). Comparatively, the groups receiving the three mixed proteins presented higher levels of these cytokines when compared to the animals immunized with the individual proteins (Fig 3A). Also, a low production of IL-4 and IL-10 was observed in all experimental groups. The ratios between IFN-γ/IL-4 and IFN-γ/IL-10 levels (Fig 3B), as well as between IL-12/IL-4 and IL-12/IL-10 levels (Fig 3C) were calculated. The obtained results were showing that mice vaccinated with the recombinant proteins plus saponin mounted a protein-specific Th1-like response. Data of immunogenicity before challenge infection that were obtained in the 2^nd^ experiment are also shown (Figure A in S1 File). In addition, the humoral response was also analyzed (Fig 4). Using SLA *L*. *infantum* as an antigen, very low responses were observed (Fig 4A), although mice vaccinated with rLiHyp1/saponin, rLiHyp6/saponin, or polyproteins/saponin had produced higher IgG2a levels than the rHRF/saponin, saponin and saline groups. When the recombinant proteins were used as antigens, all rLiHyp1/saponin, rLiHyp6/saponin, rHRF/saponin and polyproteins/saponin groups presented higher levels of protein-specific IgG2a isotype, in relation to the obtained IgG1 levels (Fig 4B). Using the OD values of each serum samples in all experimental groups, the ratio between the IgG2a and IgG1 levels was calculated, and either SLA (Fig 4C) or the recombinant proteins (Fig 4D) as antigen, a higher IgG2a production was encountered in relation to the IgG1 levels in the animals immunized with the recombinant proteins isolated or in combination, added with saponin. In this context, it could be speculated that the immunization with the recombinant proteins was able to induce a higher *in vitro* IFN-γ, IL-12 and GM-CSF production, as well as low levels of IL-4, IL-10 and parasite-specific IgG1 isotype antibodies in the vaccinated animals.

### Protective efficacy against challenge infection

**Fig 5:**
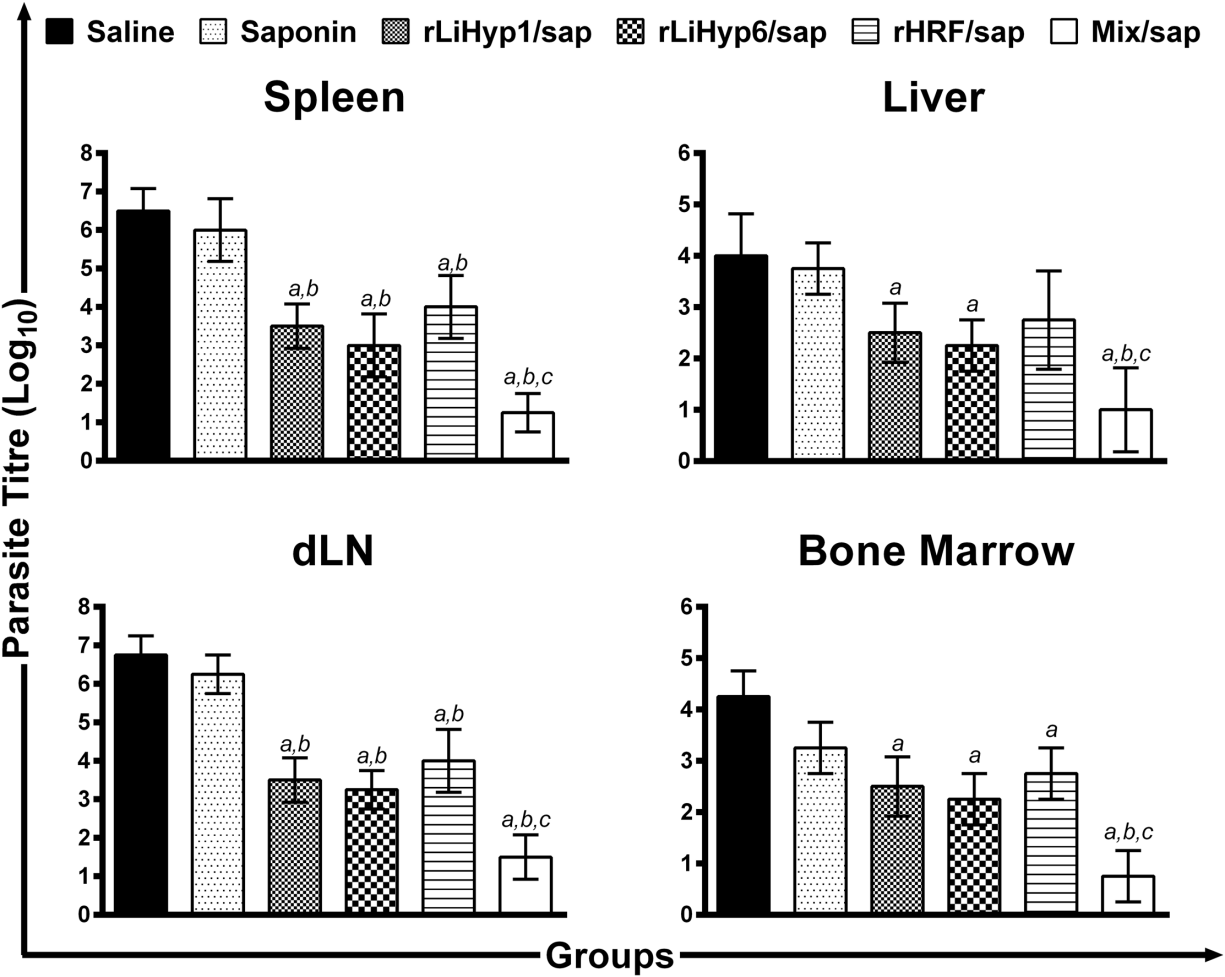
Protection of BALB/c mice against *Leishmania infantum* infection. Mice (n = 8, per group) were vaccinated subcutaneously in their left hind footpad with 25 μg of each recombinant protein (rLiHyp1, rLiHyp6, or rHRF), or with their mixture (using 25 μg of each protein), all associated with 25 μg of saponin (*Quillaja saponaria* bark saponin, Sigma). Additional mice were immunized with saponin or received saline. Three doses were administered, at two-week intervals. Four weeks after the last immunization, mice (n = 4 per group) were subcutaneously infected in the right hind footpad with 1 x 10^7^ stationary-phase promastigotes of *L*. *infantum*. Ten weeks after challenge, the parasite burden in the spleen (**A**), liver (**B**), infected paws´ draining lymph nodes (**C**), and bone marrow (**D**) were measured by a limiting-dilution technique. Bars represent the mean ± standard deviation (SD) of the groups. (*a*) indicates statistically significant difference in relation to the saline group (*P* < 0.001). (*b*) indicates statistically significant difference in relation to the saponin group (*P* < 0.001). (*c*) indicates statistically significant difference in relation to the rLiHyp1/saponin, rLiHyp6/saponin, and rHRF/saponin groups (*P* < 0.001). Data shown in this study are representative of two independent experiments, which presented similar results.

This study analyzed whether or not immunization with the recombinant proteins, administered isolately or in combination, all associated with saponin, could protect against *L*. *infantum*. The infection was followed up over a 10-week period, after which parasite burden in the spleen, liver, dLN, and BM was evaluated. In the results, significant reductions in the parasite load were observed in all evaluated organs of the vaccinated mice, when compared to those that received saline or saponin (Fig 5). In comparison to the saline group, mice vaccinated with rLiHyp1/saponin, rLiHyp6/saponin, rHRF/saponin, or with polyproteins/saponin presented reductions in the parasite load in the spleen (1.9-, 2.2-, 1.6-, and 5.2-log reductions; Fig 5A), liver (1.6-, 1.8-, 1.5-, and 4.0-log reductions; Fig 5B), dLN (2.0-, 2.1-, 1.7- and 4.5-log reductions; Fig 5C), and BM (1.7-, 1.9-, 1.6-, and 5.7-log reductions; Fig 5D), respectively. Comparing the results obtained in relation to the saponin group, it was possible to observe that mice vaccinated with rLiHyp1/saponin, rLiHyp6/saponin, rHRF/saponin, or with polyproteins/saponin presented reductions in the parasite load in the spleen (1.7-, 2.0-, 1.5-, and 4.8-log reductions; Fig 5A), liver (1.5-, 1.7-, 1.4-, and 3.8-log reductions; Fig 5B), dLN (1.8-, 1.9-, 1.6- and 4.2-log reductions; Fig 5C), and BM (1.3-, 1.4-, 1.2-, and 4.3-log reductions; Fig 5D), respectively. Evaluating comparatively the protective efficacy between the individual protein-based vaccines and polyproteins vaccine, it can be speculate that the polyproteins/saponin vaccine was able to induce a better degree of protection, due to the more significant reductions of parasite burden that were encountered in the evaluated organs in the animals of this group. Data of parasite burden obtained in the 2^nd^ experiment are also shown (Figure B in S1 File).

### Cellular response elicited after *Leishmania infantum* infection

**Fig 6:**
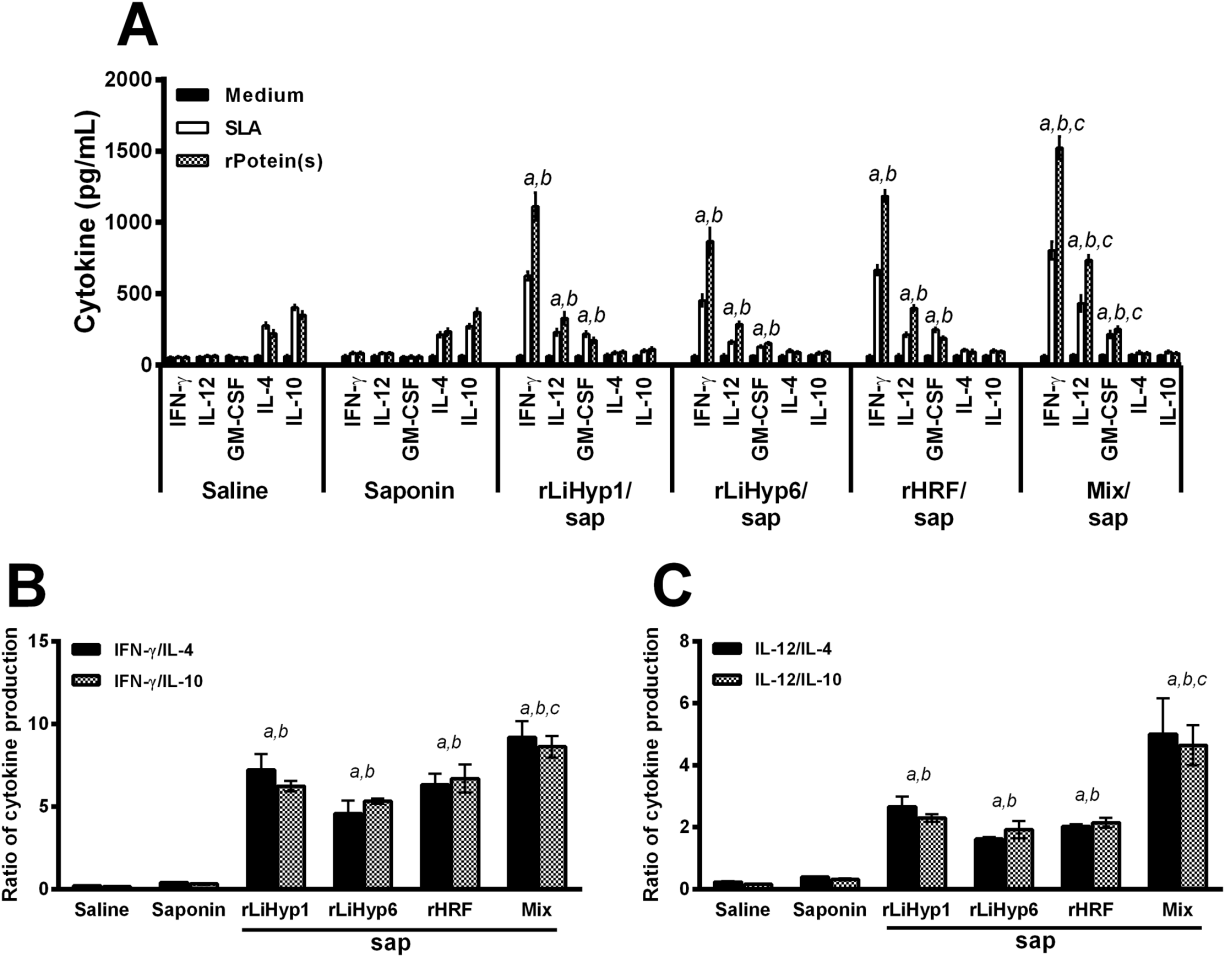
Analysis of the cellular response induced in the vaccinated animals, 10 weeks after *Leishmania infantum* infection. Mice (n = 8, per group) were vaccinated subcutaneously in their left hind footpad with 25 μg of each recombinant protein (rLiHyp1, rLiHyp6, or rHRF), or with their mixture (using 25 μg of each protein), all associated with 25 μg of saponin (*Quillaja saponaria* bark saponin, Sigma). Additional mice were immunized with saponin or received saline. Three doses were administered, at two-week intervals. Four weeks after the last immunization, mice (n = 4 per group) were subcutaneously infected using stationary-phase promastigotes of *L*. *infantum*, and 10 weeks after challenge, single cell suspensions were obtained from the spleens of the animals. Cells were incubated in RPMI 1640 medium (negative control), which was supplemented with 10% FBS, 20 mM L-glutamine, 200 U/mL penicillin, and 100 μg/mL streptomycin, at pH 7.4; or separately stimulated with rLiHyp1 (rLiHyp1/saponin group), rLiHyp6 (rLiHyp6/saponin group), rHRF (rHRF/saponin group) proteins (20 μg mL^-1^, each one); with their mixture (saline, saponin, and polyproteins/saponin groups; using 10 μg mL^-1^ of each protein), or SLA *L*. *infantum* (25 μg mL^-1^), for 48 h at 37°C in 5% CO_2_. IFN-γ, IL-12, GM-CSF, IL-4, and IL-10 levels were measured by ELISA in the culture supernatants (**A**). Bars represent the mean ± standard deviation (SD) of the groups. In addition, the ratios between IFN-γ/IL-4 and IFN-γ/IL-10 levels (**B**), as well as between IL-12/IL-4 and IL-12/IL-10 levels (**C**) were calculated, and are shown. (*a*) indicates statistically significant difference in relation to the saline group (*P* < 0.001). (*b*) indicates statistically significant difference in relation to the saponin group (*P* < 0.001). (*c*) indicates statistically significant difference in relation to the rLiHyp1/saponin, rLiHyp6/saponin, and rHRF/saponin groups (*P* < 0.001).

The production of cytokines in the supernatants of spleen cell cultures stimulated with SLA or recombinant proteins was also evaluated, 10 weeks after infection (Fig 6). In the results, spleen cells from mice vaccinated with rLiHyp1/saponin, rLiHyp6/saponin, rHRF/saponin or polyproteins/saponin produced high levels of protein- or parasite-specific IFN-γ, IL-12, and GM-CSF, in comparison to those secreted by spleen cells of animals that received saline or saponin (Fig 6A). In contrast, the protein- or parasite-driven production of IL-4 and IL-10 showed that vaccination induced no significant production of these cytokines, whereas mice from the saline and saponin groups showed a high IL-4 and IL-10 production, using both recombinant proteins and SLA in the stimulation of their spleen cells. As observed before infection, the ratios between IFN-γ/IL-4 and IFN-γ/IL-10 (Fig 6B), as well as between IL-12/IL-4 and IL-12/IL-10 (Fig 6C), using SLA to stimulate the cultures were calculated, and results showed that mice vaccinated with rLiHyp1/saponin, rLiHyp6/saponin, rHRF/saponin or polyproteins/saponin developed a parasite-specific Th1 response, after challenge infection. The polyproteins/saponin group was able to mount a more pronounced Th1 response, which was corroborated with the results obtained before challenge; indicating that this stronger immune response could contribute to the higher reduction of the parasite burden found in the vaccinated and infected animals of this group.

**Fig 7:**
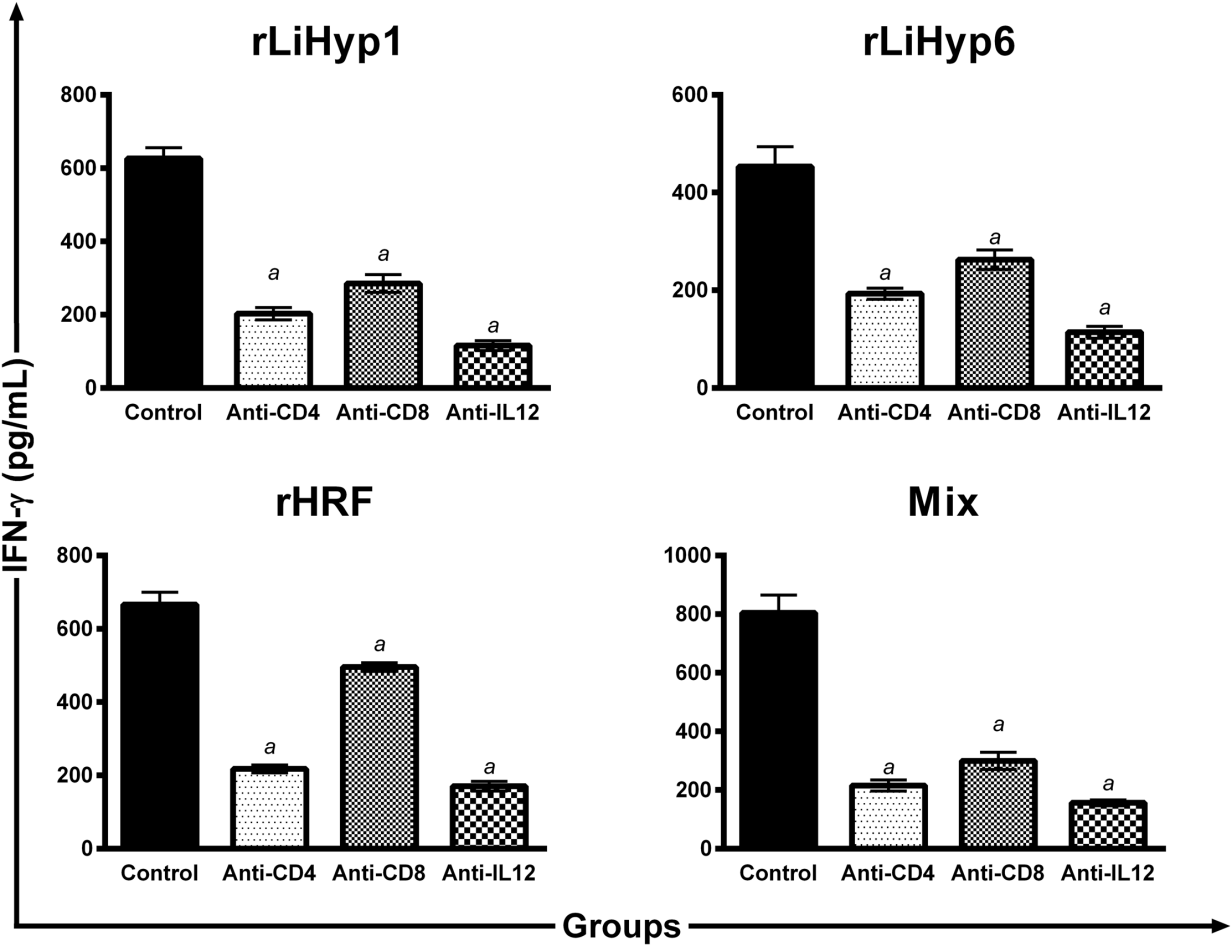
Analysis of the involvement of CD4^+^ and CD8^+^ T cells in IFN- production after *Leishmania infantum* infection. Mice (n = 8, per group) were vaccinated subcutaneously with rLiHyp1/saponin (**A**), rLiHyp6/saponin (**B**), rHRF/saponin (**C**) or polyproteins/saponin (**D**). Three doses were administered at two-week intervals. Four weeks after the last immunization, mice (n = 4 per group) were subcutaneously infected in the right hind footpad with 1 x 10^7^ stationary-phase promastigotes of *L*. *infantum*. Ten weeks after challenge, single cell suspensions were obtained from the spleens of the mice and *in vitro* stimulated with SLA (25 μg mL^-1^) for 48 h at 37°C in 5% CO_2_, in the absence (positive control) or presence of 5 μg mL^-1^ of monoclonal antibodies (mAb) against mouse IL-12, CD4, or CD8. Statistically significant differences between the positive control cells and cultures incubated with anti-CD4, anti-CD8 or anti-IL-12 monoclonal antibodies were obtained and are shown. Bars represent the mean ± standard deviation (SD) of the groups. (*a*) indicates statistically significant difference in relation to the positive control (*P* < 0.001).

The contribution of CD4^+^ and CD8^+^ T cells for the parasite-specific IFN-γ production from the spleen cells of immunized and infected mice was evaluated, 10 weeks after challenge (Fig 7). In the results, animals vaccinated with rLiHyp1/saponin (Fig 7A), rLiHyp6/saponin (Fig 7B), rHRF/saponin (Fig 7C), or with polyproteins/saponin (Fig 7D) presented a significant reduction in the IFN-γ production, when anti-IL-12, anti-CD4 or anti-CD8 monoclonal antibodies were used in the cells cultures. The addition of anti-CD8 antibodies induced a lower IFN-γ production in the rLiHyp1/saponin and rLiHyp6/saponin groups (Fig 7A and 7B, respectively), in comparison to the rHRF/saponin group (Fig 7C), possibly due to the fact that these hypothetical proteins were found in the amastigote stage of the parasites. In the polyproteins/saponin group, the reduction of the IFN-γ production was achieved when both anti-CD4 and anti-CD8 monoclonal antibodies were used in the cells cultures (Fig 7D). Data of immunogenicity after challenge infection that were obtained in the 2^nd^ experiment are also shown (Figures C, D and E in S1 File).

### Humoral response and nitrite production induced after *Leishmania infantum* infection

**Fig 8:**
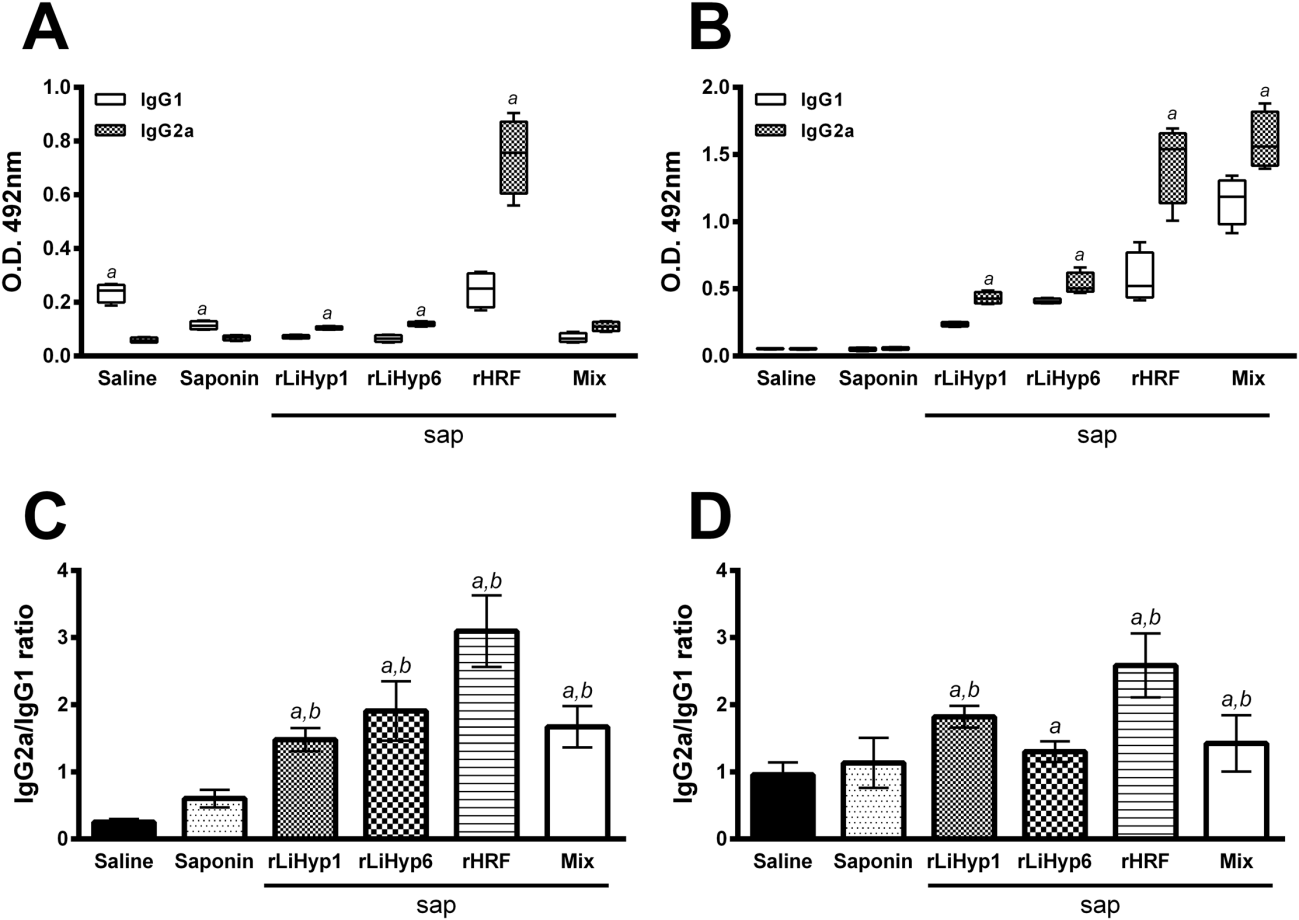
Humoral response induced in the vaccinated and infected BALB/c mice, 10 weeks after *Leishmania infantum* infection. Mice (n = 8, per group) were vaccinated subcutaneously with rLiHyp1/saponin, rLiHyp6/saponin, rHRF/saponin or polyproteins/saponin. Three doses were administered at two-week intervals. Four weeks after the last immunization, mice (n = 4 per group) were subcutaneously infected using stationary-phase promastigotes of *L*. *infantum*, and 10 weeks after challenge, sera samples were obtained from the animals to evaluate the humoral response. The reactivity based on IgG1 and IgG2a isotypes represented by whisker (min to max) plots against SLA (**A**) and the respective recombinant proteins (**B**) are shown. In each group vaccinated with the recombinant protein, the respective antigen was used. In the saline and saponin groups, as well as in the polyproteins/saponin group, the protein mixture was used as antigen. Also, SLA was used as antigen in all groups. For the assays, 1.0, 1.0, and 0.5 μg per well of rLiHyp1, rLiHyp6, and rHRF proteins, respectively; 0.5 μg per well of each protein composing the antigenic mixture, and 2.0 μg per well of SLA *L*. *infantum*, were used in the ELISA assays. The sera samples were diluted at 1:200, and the anti-mouse IgG1 and IgG2a horseradish-peroxidase conjugated antibodies were employed in a 1:5,000 and 1:10,000 dilution, respectively. The ratios between the IgG2a/IgG1 levels against SLA (**C**) and the respective recombinant proteins (**D**) were calculated, and are also shown. Bars represent the mean ± standard deviation (SD) of the groups. (*a*) indicates statistically significant difference in relation to the saline group (*P* < 0.001). (*b*) indicates statistically significant difference in relation to the saponin group (*P* < 0.001).

Evaluating the antibody production in the vaccinated and infected animals (Fig 8), 10 weeks after infection, it was possible to verify that animals vaccinated with rLiHyp1/saponin, rLiHyp6/saponin, rHRF/saponin, or polyproteins/saponin presented higher levels of parasite- and protein-specific IgG2a antibodies in relation to the IgG1 levels (Fig 8A and 8B, respectively). The rHRF/saponin group presented the higher levels of both IgG1 and IgG2a levels, although the IgG2a production has been significantly higher than IgG1 levels. The ratio between the IgG2a and IgG1 levels using SLA (Fig 8C) or recombinant proteins (Fig 8D) as antigens was also calculated, and it was possible to observe that all vaccinated groups presented higher IgG2a/IgG1 values, when compared to the saline and saponin groups.

**Fig 9:**
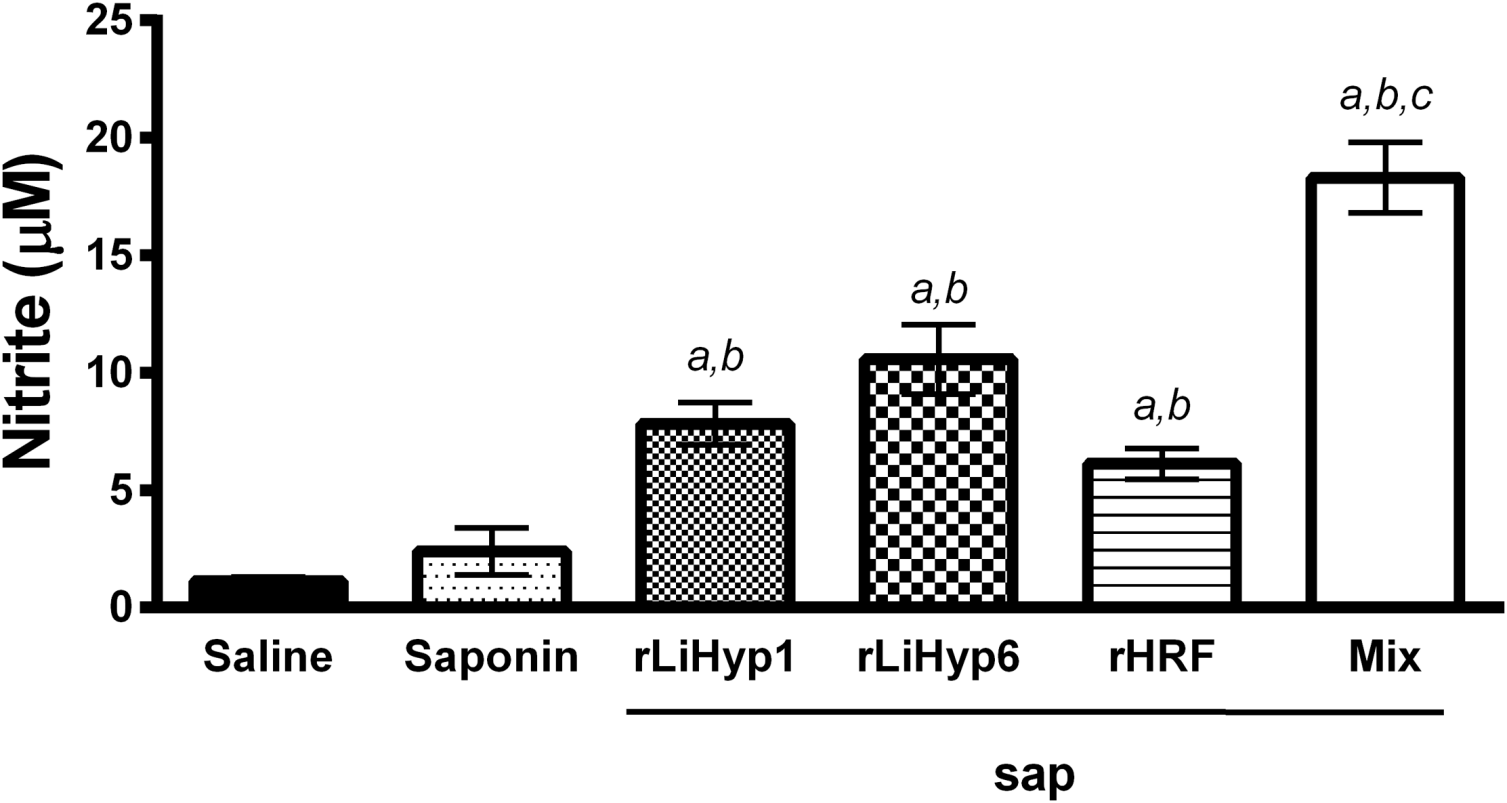
Nitric oxide production by vaccinated and infected animals, 10 weeks after challenge infection. Mice (n = 8, per group) were vaccinated subcutaneously with rLiHyp1/saponin, rLiHyp6/saponin, rHRF/saponin or polyproteins/saponin. Three doses were administered at two-week intervals. Four weeks after the last immunization, mice (n = 4 per group) were subcutaneously infected in the right hind footpad with 1 x 10^7^ stationary-phase promastigotes of *L*. *infantum*. Ten weeks after challenge, single cell suspensions were obtained from the spleens of the animals and incubated in RPMI 1640 medium (negative control), which was supplemented with 10% FBS, 20 mM L-glutamine, 200 U/mL penicillin, and 100 μg/mL streptomycin, at pH 7.4; or stimulated with SLA *L*. *infantum* (25 μg mL^-1^), for 48 h at 37°C in 5% CO_2_. Then, the nitrite production was evaluated in the culture supernatant using a nitric oxide assay kit, and the results are expressed in μM. Bars represent the mean ± standard deviation (SD) of the groups. (*a*) indicates statistically significant difference in relation to the saline group (*P* < 0.001). (*b*) indicates statistically significant difference in relation to the saponin group (*P* < 0.001). (*c*) indicates statistically significant difference in relation to the rLiHyp1/saponin, rLiHyp6/saponin, and rHRF/saponin groups (*P* < 0.001).

In an attempt to determine the influence of the immunizations of the different vaccines on the *L*. *infantum* specific killing effectors functions, the nitrite concentration (employed as an indicator of NO production) was analyzed in the culture supernatants after an *in vitro* stimulation using parasite antigens (Fig 9). In the results, the nitrite production was significantly higher in the mice vaccinated with rLiHyp1/saponin, rLiHyp6/saponin, rHRF/saponin, or polyproteins/saponin, when compared to the saline and saponin groups. The highest level on nitrite in supernatants was observed in cultures established form mice inoculated with the polyproteins/saponin vaccine.

## Discussion

It has been showed that *Leishmania spp*. proteins that react with antibodies from VL dogs could be associated with antigenicity and protective responses, representing potential diagnostic markers and vaccine candidates against leishmaniasis [5,11]. As described in detail previously [30], amastigote antigens have been far less evaluated as vaccine candidates against VL. One could speculate that this could be due to the fact that promastigotes are more easily cultured *in vitro*, as opposed to amastigotes; thus hampering the identification of amastigote-specific antigens [13,30,38]. However, a vaccine that is able to elicit a protective immune response against amastigote proteins could present advantages for both prophylactic and therapeutic conditions. Also, in contrast to promastigote forms, the amastigotes reside within host cells and are targets for CD8^+^ T cells, elements involved in the protection against *Leishmania spp*. infection. In the present study, the DNA encoding of two hypothetical proteins, which were recently identified expressed in the amastigote stage of *L*. *infantum*, were cloned and combined with a promastigote protein; with the purpose of composing a polyproteins vaccine to be evaluated against VL. Also, due to their recognition by antibodies of VL dogs, these proteins were evaluated for the serodiagnosis of CVL.

Vaccine studies concerning leishmaniasis call for the critical evaluation of the parasitological and immunological parameters in murine models [13,28]. To properly evaluate new antigens, it is important to optimize their dose and route of administration [39]. The present study aimed to evaluate a polyproteins vaccine composed by three different antigens, based on their potential to induce a more effective protective immunity against murine VL. In relation to the immunogenic composition, some authors have used smaller amounts of single proteins in a 1:1:1 ratio, in the attempt to evaluate the efficacy of their vaccine candidates [40,41]. Taking this into account, our group chose to compose the polyproteins vaccine with the same amount of protein in a fixed ratio of 1:1:1. Nonetheless, it was possible to verify that the polyproteins vaccine plus saponin was able to induce a more pronounced Th1 response in the vaccinated animals, which was based on *in vitro* protein- and parasite-specific production of IFN-γ, IL-12, and GM-CSF, combined with the presence of low levels of IL-4 and IL-10 in the spleen cells of the immunized animals, as well as by low levels of protein- and parasite-specific IgG1 isotype antibodies. The results of immunogenicity proved to be better when the polyproteins vaccine, as compared to the employ of individual proteins, was used. In addition, after infection, mice immunized with the polyproteins vaccine plus saponin displayed more significant reductions in the parasite burden in all evaluated organs, which were correlated with a parasite-dependent IFN-γ production in the spleen of the animals, which is one of the main cytokines implicated in the acquired immunity against VL [16,42].

The evaluation of parasite burden was performed at week 10 after infection, since in the murine model employed; high numbers of parasites are present in different organs in this period of time [43]. In the absence of parasite determination at longer times after challenge, it cannot be discarded that vaccinated mice can present a delay in parasite spreading. Further investigation should be made to study parasite evolution in different organs, since mice infected with *L*. *infantum* have organ specific responses that destroy parasites in the liver, while they become chronic in the spleen. Like demonstrated here, the development and design of combining-protein vaccines has showed an improved protective efficacy against VL. In a recent study, a vaccine containing *L*. *tarentolae* expressing the *L*. *donovani* A2 protein along with cysteine proteinases (CPA and CPB) was evaluated in the protection of BALB/c mice against *L*. *infantum* infection. The authors showed that the combined vaccine induced a protective immunity, which was based on a Th1 response with high levels of IFN-γ prior and after challenge, as well as by low levels of IL-10 produced after infection. Moreover, the protection was correlated with a high nitric oxide production, and a low parasite burden in the vaccinated and infected animals was observed in this study [44]. In other work, the efficacy of a combined vaccine using the same system, live recombinant *L*. *tarentolae* expressing cysteine proteinases (CPA and CPB), but added with PpSP15, an immunogenic salivary protein from *Phlebotomus papatasi*; was also protective against *L*. *infantum*, being this protection also based on the development of a Th1 immune response in the vaccinated and infected animals [45].

Evaluating the involvement of the T cells in the immune response induced after infection in the present study, CD4^+^ T cells proved to be the major source of IFN-γ in the protected mice, since depletion of these cells in cultures of spleen cells stimulated with SLA *L*. *infantum* significantly reduced this response. In relation to the involvement of CD8^+^ T cells, although the IFN-γ production had been also diminished in the vaccinated and infected animals, this production was higher in relation to that obtained when the anti-CD4 monoclonal antibody was used. Previous reports have shown that the activation of both CD4^+^ and CD8^+^ T cells subsets is important for the killing of parasites in mice vaccinated with *Leishmania spp*. recombinant proteins [13,20,46]; although the CD4^+^ T cells response had been more important in the IFN-γ production, as well as in the induction of protection against challenge infection [13]. However, although the results of the present study have showed a discrimination in the IFN-γ production in the spleen cells cultures after using anti-CD4 and anti-CD8 monoclonal antibodies, this results could be considered a proof-of-concept of the involvement of the T cells, once these cells were not separate of others, such as macrophages in the *in vitro* cultures; being then this production considered as an indirect implication of the involvement of CD4^+^ and CD8^+^ T cells in the IFN-γ production.

Spleen cells derived from the vaccinated mice, when compared to the control groups, produced higher levels of protein- and parasite-specific GM-CSF, a cytokine linked to macrophage activation and resistance of murine models against infection caused by some *Leishmania spp*. species, such as *L*. *infantum* [13,17,20], *L*. *major* [47], and *L*. *donovani* [48]. GM-CSF plays also with an important role in activation and functional maturation of dendritic cells [49]. Moreover, it can recruits cells, such as monocytes and neutrophils, to the *Leishmania spp*. infection site, and contributing to the development of an effective immune response. It has been also used as an adjuvant in vaccine candidates for malaria [50], HIV [51], leishmaniasis [52], and others. These studies have shown that GM-CSF significantly increases the immunogenicity of the evaluated antigens, contributing to the protection against disease. The present study showed that the polyproteins vaccine plus saponin induced a low production of IL-4 and IL-10 before infection, which was maintained in low levels after challenge. On the other hand, animals from the control (saline and saponin) groups showed a significantly higher production of these cytokines after *L*. *infantum* infection. Indeed, the control of the parasite-mediated IL-10 response in mice may be important for protection against visceral disease, since IL-10 is considered to be the most important factor for disease progression caused by viscerotropic *Leishmania spp*. species, like described in both IL-10 deficient mice [17,53,54], and in mice treated with an anti-IL-10 receptor antibody [55].

As described in detail previously [13], in BALB/c mice, the IL-4-dependent production of IgG1 isotype antibodies is associated with disease progression caused by *L*. *infantum* [13,20,36], and *L*. *amazonensis* [34,56]. For instance, in studies of immunization of BALB/c mice with the recombinant A2 protein [16,34] or *Leishmania* ribosomal proteins [17] plus saponin, the animals were protected against infection, being this protection correlated with a decrease in parasite-specific IL-4 and IL-10 mediated response, as well as by low levels of parasite-specific IgG1 isotype antibodies. In this context, in the present study, mice that received saline or saponin and that were not protected against infection, presented high parasite-specific IgG1 antibody levels, when compared to their IgG2a levels. An important immunological control of intracellular parasitism is based on the production of oxygen derivative metabolites [57]. Therefore, the present study investigated the NO production by spleen cells of the vaccinated and infected animals, 10 weeks after infection. It was observed that the protected animals, which were immunized with the recombinant proteins isolate or in combination, produced higher levels of NO, when compared to the control groups. According to these data, it could be speculated a possible activation of an anti-parasite effectors mechanisms mediated by NO, as previously described by others [17,58,59].

Serological tests are currently recommended for the laboratorial diagnosis of CVL. Some of them, such as IFAT and ELISA, are used as diagnostic markers; however, their low sensitivity to detect cases from animals with low levels of antileishmanial antibodies, as well as by their specificity hampered due to cross-reactivity with antibodies of animals presenting other pathologies, such as Chagas´ disease, babesiosis and erlichiose; are problems to be solved [60,61]. In Brazil, there are two commercial vaccines against CVL, Leishmune^®^ [62], and Leish-Tec^®^ [16]. However, although protective, these vaccines can induce seroconversion in the immunized animals, causing them to be classified as false-positive in the serological assays performed [63–65]. In the present study, it was possible to verify that all three evaluated recombinant proteins were specifically recognized by antibodies of symptomatic and asymptomatic VL dogs, and the results of sensitivity and specificity values were better in comparison to data obtained using an antigenic preparation of the parasites. In addition, the serological results obtained with the rLiHyp1 protein are in accordance with previous data [13]. Moreover, the ability of these antigens to distinguish the CVL sera from those from non-infected animals living in endemic and non-endemic areas of leishmaniasis, leads to the possibility of reducing the number of false-positive results usually encountered when these sera are evaluated in the serological assays performed [66]. However, the number and variety of sera used in the present study could be considered a limiting factor of the serological assays. In this context, data here presented should be taken as a proof-of-concept of the efficacy of these proposed antigens to be employed in the serodiagnosis of CVL, and may well serve as a reference for further assays. On the other hand, we believe that, after an adequate validation, the rLiHyp1, rLiHyp6 and rHRF proteins may be promptly applied for a sensitive and specific serodiagnosis of CVL.

In recent years, advances in the development of vaccines against VL have been based on molecularly defined antigens [13,16,19,27,29,67]. Although second generation vaccines are currently being tested in clinical trials, the screening of new candidates will help to further increase the prophylactic efficacy of effective candidates against this disease. It has been proposed that a combination of different parasite proteins leading to the development of polyproteins vaccines could help to produce a more robust and effective prophylactic vaccine, presenting more protective characteristics [1,43,44]. The WHO has postulated that multiproteic vaccines could present a more protective efficacy against *Leishmania spp*. infection [1]. In addition, to be effective as a vaccine candidate against leishmaniasis, its components should either be shared by different parasite species, or be based on antigens expressed in both parasite stages. The use of chimeric vaccines combining multi-proteins and/or poly-epitopes may well provide benefits in terms of simplicity and production costs, since only one vaccine would be produced, besides of it could be protective against different *Leishmania spp*. species [68].

Although offering advantages in terms of safety and production´ costs, protein-based vaccines are less immunogenic and must be supplemented with immune adjuvants, in order to boost their immunogenicity [69]. In this context, for the development of a successful vaccine, one could speculate that the association of an effective adjuvant will be desirable. Recently, the rLiHyp1 protein was evaluated as a vaccine candidate against *L*. *infantum* infection, being associated with two polysaccharide-rich fractions (F2 and F4 fractions), which were derived from *Agaricus blazei* mushroom. The authors showed that the immunization using the rLiHyp1 protein plus F2 or F4 fractions of *A*. *blazei* was able to induce protection against challenge infection, which was based on the development of a Th1 immune response in the vaccinated and infected animals [70]. In the same study, the rLiHyp1 protein was administered isolated, and the results showed that it was not protective without the association of an immune adjuvant.

Thus, the data of the present study could be taken as a proof-of-concept of the efficacy from a developed polyproteins vaccine that, when administered in association with an adjuvant, could be used in the protection against VL. Additional studies evaluating this polyproteins vaccine against other *Leishmania spp*. species, such as against species causing tegumentary leishmaniasis; could be performed. In relation to their diagnostic performance, the rLiHyp1, rLiHyp6 and rHRF proteins could be also evaluated in future studies as new diagnostic markers for the CVL.

## Supporting Information

S1 File Immunological and parasitological parameters evaluated in the vaccinated animals, before and after the *Leishmania infantum* challenge infection. Mice (n = 8, per group) were vaccinated subcutaneously in their left hind footpad with 25 g of each recombinant protein (rLiHyp1, rLiHyp6, or rHRF), or with their mixture (using 25 g of each protein), all associated with 25 g of saponin (*Quillaja saponaria* bark saponin, Sigma). Additional mice were immunized with saponin or received saline. Three doses were administered, at two-week intervals. Four weeks after the last immunization, mice (n = 4 per group) were subcutaneously infected in the right hind footpad with 1 x 10^7^ stationary-phase promastigotes of *L*. *infantum*. Before and 10 weeks after challenge, the cytokine levels were evaluated. For this, cells were incubated in complete RPMI 1640 medium (negative control), or separately stimulated with rLiHyp1 (rLiHyp1/saponin group), rLiHyp6 (rLiHyp6/saponin group), rHRF (rHRF/saponin group) proteins (20 g mL^-1^, each one); with their mixture (saline, saponin, and polyproteins/saponin groups; using 10 g mL^-1^ of each protein), or SLA *L*. *infantum* (25 μg mL^-1^), for 48 h at 37°C in 5% CO_2_. IFN-, IL-12, GM-CSF, IL-4, and IL-10 levels were measured by ELISA in the culture supernatants (**Figure A**, before and **Figure D**, after infection). Also 10 weeks after challenge, the parasite burden was determined in the spleen, liver, infected paws´ draining lymph nodes, and bone marrow of the animals (**Figure B**). Using the supernatants of the SLA *L*. *infantum*-stimulated spleen cells, the nitrite production was evaluated in this time (**Figure C**). (*a*) indicates statistically significant difference in relation to the saline group (*P* < 0.001). (*b*) indicates statistically significant difference in relation to the saponin group (*P* < 0.001). (*c*) indicates statistically significant difference in relation to the rLiHyp1/saponin, rLiHyp6/saponin, and rHRF/saponin groups (*P* < 0.001). To evaluate the involvement of CD4^+^ and CD8^+^ T cells in IFN- production after *L*. *infantum* infection, single cell suspensions that were stimulated with SLA (25 g mL^-1^) were incubated in the absence (positive control) or presence of 5 g mL^-1^ of monoclonal antibodies (mAb) against mouse IL-12, CD4, or CD8 (**Figure E**). (*a*) Statistically significant differences between the positive control cells and cultures incubated with anti-CD4, anti-CD8 or anti-IL-12 monoclonal antibodies were obtained and are shown (*P* < 0.001). In all panels, bars represent the mean ± standard deviation (SD) of the groups.
(TIF)

## References

[1] World Health Organization (2010) Control of the leishmaniases. World Health Organ Tech Rep Ser: 22–26. 21485694

[2] Croft SL, Coombs GH (2003) Leishmaniasis: current chemotherapy and recent advances in the search for novel drugs. Trends Parasitol 19: 502–508. 14580961 10.1016/j.pt.2003.09.008

[3] Minodier P, Parola P (2007) Cutaneous leishmaniasis treatment. Travel Med Infect Dis 5: 150–158. 10.1016/j.tmaid.2006.09.004 17448941

[4] Oliveira LF, Schubach AO, Martins MM, Passos SL, Oliveira RV, Marzochi MC, et al (2011) Systematic review of the adverse effects of cutaneous leishmaniasis treatment in the New World. Acta Trop 118: 87–96. 10.1016/j.actatropica.2011.02.007 21420925

[5] Coelho VTS, Oliveira JS, Valadares DG, Chávez-Fumagalli MA, Duarte MC, Lage PS, et al (2012) Identification of proteins in promastigote and amastigote-like Leishmania using an immunoproteomic approach. PLoS Negl Trop Dis 6: 1–10. 10.1371/journal.pntd.0001430 PMC3260309 22272364

[6] Gramiccia M, Gradoni L (2005) The current status of zoonotic leishmaniases and approaches to disease control. Int J Parasitol 35: 1169–1180. 10.1016/j.ijpara.2005.07.001 16162348

[7] Miró G, Cardoso L, Pennisi MG, Oliva G, Baneth G (2008) Canine leishmaniosis—new concepts and insights on an expanding zoonosis: part two. Trends Parasitol 24: 371–377. 10.1016/j.pt.2008.05.003 18603476

[8] Barbiéri CL (2006) Immunology of canine leishmaniasis. Parasite Immunol 28: 329–337. 10.1111/j.1365-3024.2006.00840.x 16842269

[9] Ciaramella P, Oliva G, Luna R, Ambrosio R, Cortese L, Cortese L, et al (1997) A retrospective clinical study of canine leishmaniasis in 150 dogs naturally infected by Leishmania infantum. Vet Rec 141: 539–543. 10.1136/vr.141.21.539 9413121

[10] Garcia-Alonso M, Nieto CG, Blanco A, Requena JM, Alonso C, Navarrete I (1996) Presence of antibodies in the aqueous humour and cerebrospinal fluid during Leishmania infections in dogs. Pathological features at the central nervous system. Parasite Immunol 18: 539–546. 9226692 10.1046/j.1365-3024.1996.d01-28.x

[11] Alves WA, Bevilacqua PD (2004) Reflexões sobre a qualidade do diagnóstico da leishmaniose visceral canina em inquéritos epidemiológicos: o caso da epidemia de Belo Horizonte, Minas Gerais, Brasil, 1993–1997. Cad Saude Publica 20: 259–265. 10.1590/S0102-311X2004000100043 15029328

[12] Miró G, Montoya A, Mateo M, Alonso A, García S, García A, et al (2007) A leishmaniosis surveillance system among stray dogs in the region of Madrid: Ten years of serodiagnosis (1996–2006). Parasitol Res 101: 253–257. 10.1007/s00436-007-0497-8 17323100

[13] Martins VT, Chávez-Fumagalli MA, Costa LE, Martins AMCC, Lage PS, Lage DP, et al (2013) Antigenicity and protective efficacy of a Leishmania amastigote-specific protein, member of the super-oxygenase family, against visceral leishmaniasis. PLoS Negl Trop Dis 7: e2148 10.1371/journal.pntd.0002148 23573301 PMC3610918

[14] Baharia RK, Tandon R, Sharma T, Suthar MK, Das S, Siddiqi MI, et al (2015) Recombinant NAD-dependent SIR-2 protein of Leishmania donovani: immunobiochemical characterization as a potential vaccine against visceral leishmaniasis. PLoS Negl Trop Dis 9: e0003557 10.1371/journal.pntd.0003557 25745863 PMC4351947

[15] Afonso LC, Scott P (1993) Immune responses associated with susceptibility of C57BL/10 mice to Leishmania amazonensis. Infect Immun 61: 2952–2959. 8514400 10.1128/iai.61.7.2952-2959.1993 PMC280944

[16] Blackwell JM (1996) Genetic susceptibility to leishmanial infections: studies in mice and man. Parasitology 112 Suppl: S67–S74. 8684837

[17] Fernandes AP, Costa MMS, Coelho EAF, Michalick MSM, Freitas E, Melo MN, et al (2008) Protective immunity against challenge with Leishmania (Leishmania) chagasi in beagle dogs vaccinated with recombinant A2 protein. Vaccine 26: 5888–5895. 18786587 10.1016/j.vaccine.2008.05.095

[18] Chávez-Fumagalli MA, Costa MAF, Oliveira DM, Ramírez L, Costa LE, Duarte MC, et al (2010) Vaccination with the Leishmania infantum ribosomal proteins induces protection in BALB/c mice against Leishmania chagasi and Leishmania amazonensis challenge. Microbes Infect 12: 967–977. 10.1016/j.micinf.2010.06.008 20601076

[19] Das A, Ali N (2012) Vaccine prospects of killed but metabolically active Leishmania against visceral leishmaniasis. Expert Rev Vaccines 11: 783–785. Available: http://www.ncbi.nlm.nih.gov/pubmed/22913255. 10.1586/erv.12.50 22913255

[20] Ramírez L, Santos DM, Souza AP, Coelho EAF, Barral A, Alonso C, et al (2013) Evaluation of immune responses and analysis of the effect of vaccination of the Leishmania major recombinant ribosomal proteins L3 or L5 in two different murine models of cutaneous leishmaniasis. Vaccine 31: 1312–1319. 10.1016/j.vaccine.2012.12.071 23313653

[21] Costa LE, Goulart LR, Jesus-Pereira NC, Lima MI, Duarte MC, et al (2014) Mimotope-based vaccines of Leishmania infantum antigens and their protective efficacy against visceral leishmaniasis. PLoS One 9: e110014 10.1371/journal.pone.0110014 25333662 PMC4198211

[22] Green SJ, Mellouk S, Hoffman SL, Meltzer MS, Nacy CA (1990) Cellular mechanisms of nonspecific immunity to intracellular infection: cytokine-induced synthesis of toxic nitrogen oxides from L-arginine by macrophages and hepatocytes. Immunol Lett 25: 15–19. 10.1016/0165-2478(90)90083-3 2126524

[23] Wilson ME, Jeronimo SMB, Pearson RD (2005) Immunopathogenesis of infection with the visceralizing Leishmania species. Microb Pathog 38: 147–160. 10.1016/j.micpath.2004.11.002 15797810

[24] Stäger S, Alexander J, Carter KC, Brombacher F, Kaye PM (2003) Both interleukin-4 (IL-4) and IL-4 receptor α signaling contribute to the development of hepatic granulomas with optimal antileishmanial activity. Infect Immun 71: 4804–4807. 10.1128/IAI.71.8.4804-4807.2003 12874364 PMC166035

[25] Joshi J, Kaur S (2014) Studies on the protective efficacy of second-generation vaccine along with standard antileishmanial drug in Leishmania donovani infected BALB/c mice. Parasitology 141: 554–562. Available: http://www.ncbi.nlm.nih.gov/pubmed/24618257. 10.1017/S0031182013001959 24618257

[26] Kharazmi A, Kemp K, Ismail A, Gasim S, Gaafar A, Kurtzhals JA, et al (1999) T-cell response in human leishmaniasis. Immunology Letters 65: 105–108. 10.1016/S0165-2478(98)00132-1 10065635

[27] Stäger S, Smith DF, Kaye PM (2000) Immunization with a recombinant stage-regulated surface protein from Leishmania donovani induces protection against visceral leishmaniasis. J Immunol 165: 7064–7071. 11120835 10.4049/jimmunol.165.12.7064

[28] Iborra S, Parody N, Abánades DR, Bonay P, Prates D, Novais FO, et al (2008) Vaccination with the Leishmania major ribosomal proteins plus CpG oligodeoxynucleotides induces protection against experimental cutaneous leishmaniasis in mice. Microbes Infect 10: 1133–1141. 10.1016/j.micinf.2008.06.002 18603012

[29] Agallou M, Smirlis D, Soteriadou KP, Karagouni E (2012) Vaccination with Leishmania histone H1-pulsed dendritic cells confers protection in murine visceral leishmaniasis. Vaccine 30: 5086–5093. 10.1016/j.vaccine.2012.05.075 22704924

[30] Fernandes AP, Coelho EAF, Machado-Coelho GLL, Grimaldi G, Gazzinelli RT (2012) Making an anti-amastigote vaccine for visceral leishmaniasis: Rational, update and perspectives. Curr Opin Microbiol 15: 476–485. 22698479 10.1016/j.mib.2012.05.002

[31] Wenzel UA, Bank E, Florian C, Forster S, Zimara N, Steinacker J, et al (2012) Leishmania major parasite stage-dependent host cell invasion and immune evasion. FASEB J 26: 29–39. 10.1096/fj.11-184895 21908716

[32] Kang HS, Lee MJ, Song H, Han SH, Kim YM, Im JI, et al (2001) Molecular identification of IgE-dependent histamine-releasing factor as a B cell growth factor. J Immunol 166: 6545–6554. 11359806 10.4049/jimmunol.166.11.6545

[33] Kang J-A, Kim W-S, Park S-G (2014) Notch1 is an important mediator for enhancing of B-cell activation and antibody secretion by Notch ligand. Immunology 143: 550–559. 10.1111/imm.12333 24913005 PMC4253503

[34] Coelho EAF, Tavares CAP, Carvalho FAA, Chaves KF, Teixeira KN, Rodrigues RC, et al (2003) Immune responses induced by the Leishmania (Leishmania) donovani A2 antigen, but not by the LACK antigen, are protective against experimental Leishmania (Leishmania) amazonensis infection. Infect Immun 71: 3988–3994. 10.1128/IAI.71.7.3988-3994.2003 12819086 PMC162020

[35] Bradford MM (1976) A rapid and sensitive method for the quantitation of microgram quantities of protein utilizing the principle of protein-dye binding. Anal Biochem 72: 248–254. 10.1016/0003-2697(76)90527-3 942051

[36] Zanin FHC, Coelho EAF, Tavares CAP, Marques-da-Silva EA, Costa MMS, Rezende SA, et al (2007) Evaluation of immune responses and protection induced by A2 and nucleoside hydrolase (NH) DNA vaccines against Leishmania chagasi and Leishmania amazonensis experimental infections. Microbes Infect 9: 1070–1077. 10.1016/j.micinf.2007.05.012 17644455

[37] Green LC, Wagner DA, Glogowski J, Skipper PL, Wishnok JS, Tannenbaum SR (1982) Analysis of nitrate, nitrite, and [15N]nitrate in biological fluids. Anal Biochem 126: 131–138. 10.1016/0003-2697(82)90118-X 7181105

[38] Kaur S, Kaur T, Garg N, Mukherjee S, Raina P, Athokpam V (2008) Effect of dose and route of inoculation on the generation of CD4^+^ Th1/Th2 type of immune response in murine visceral leishmaniasis. Parasitol Res 103: 1413–1419. 10.1007/s00436-008-1150-x 18751727

[39] Galli V, Simionatto S, Marchioro SB, Klabunde GHF, Conceição FR, Dellagostin AO (2013) Recombinant secreted antigens from Mycoplasma hyopneumoniae delivered as a cocktail vaccine enhance the immune response of mice. Clin Vaccine Immunol 20: 1370–1376. 10.1128/CVI.00140-13 23803903 PMC3889581

[40] Tsenova L, Harbacheuski R, Moreira AL, Ellison E, Dalemans W, Alderson MR, et al (2006) Evaluation of the Mtb72F polyprotein vaccine in a rabbit model of tuberculous meningitis. Infect Immun 74: 2392–2401. 16552069 10.1128/IAI.74.4.2392-2401.2006 PMC1418915

[41] Resende DM, Caetano BC, Dutra MS, Penido MLO, Abrantes CF, Verly RM, et al (2008) Epitope mapping and protective immunity elicited by adenovirus expressing the Leishmania amastigote specific A2 antigen: correlation with IFN-γ and cytolytic activity by CD8^+^ T cells. Vaccine 26: 4585–4593. 18588933 10.1016/j.vaccine.2008.05.091

[42] Rachamim N, Jaffe CL (1993) Pure protein from Leishmania donovani protects mice against both cutaneous and visceral leishmaniasis. J Immunol 150: 2322–2331. 8450215

[43] Oliveira DM, Valadares DG, Duarte MC, Costa LE, Martins VT, Costa LE, et al (2012) Evaluation of parasitological and immunological parameters of *Leishmania chagasi* infection in BALB/c mice using different doses and routes of inoculation of parasites. Parasitol Res 110: 1277–1285. 10.1007/s00436-011-2628-5 21915627

[44] Saljoughian N, Taheri T, Zahedifard F, Taslimi Y, Doustdari F, Bolhassani A, et al (2013) Development of novel prime-boost strategies based on a tri-gene fusion recombinant L. tarentolae vaccine against experimental murine visceral leishmaniasis. PLoS Negl Trop Dis 7: 1–15. 10.1371/journal.pntd.0002174 PMC3630202 23638195

[45] Zahedifard F, Gholami E, Taheri T, Taslimi Y, Doustdari F, Seyed N, et al (2014) Enhanced protective efficacy of nonpathogenic recombinant Leishmania tarentolae expressing cysteine proteinases combined with a sand fly salivary antigen. PLoS Negl Trop Dis 8 10.1371/journal.pntd.0002751 PMC3967951 24675711

[46] Bhowmick S, Ravindran R, Ali N (2008) Gp63 in stable cationic liposomes confers sustained vaccine immunity to susceptible BALB/c mice infected with Leishmania donovani. Infect Immun 76: 1003–1015. 10.1128/IAI.00611-07 18195029 PMC2258822

[47] Dumas C, Muyombwe A, Roy G, Matte C, Ouellette M, Olivier M, et al (2003) Recombinant Leishmania major secreting biologically active granulocyte-macrophage colony-stimulating factor survives poorly in macrophages in vitro and delays disease development in mice. Infect Immun 71: 6499–6509. 10.1128/IAI.71.11.6499-6509.2003 14573672 PMC219543

[48] Murray HW, Cervia JS, Hariprashad J, Taylor AP, Stoeckle MY, Hockman H (1995) Effect of granulocyte-macrophage colony-stimulating factor in experimental visceral leishmaniasis. J Clin Invest 95: 1183–1192. 10.1172/JCI117767 7883967 PMC441456

[49] Bayih AG, Daifalla NS, Gedamu L (2014) DNA-protein immunization using Leishmania peroxidoxin-1 induces a strong CD4^+^ T cell response and partially protects mice from cutaneous leishmaniasis: role of fusion murine granulocyte-macrophage colony-stimulating factor DNA adjuvant. PLoS Negl Trop Dis 8: e3391 10.1371/journal.pntd.0003391 25500571 PMC4263403

[50] Weiss WR, Ishii KJ, Hedstrom RC, Sedegah M, Ichino M, Barnhart K, et al (1998) A plasmid encoding murine granulocyte-macrophage colony-stimulating factor increases protection conferred by a malaria DNA vaccine. J Immunol 161: 2325–2332. 9725227

[51] Lai L, Kwa S, Kozlowski PA, Montefiori DC, Ferrari G, Johnson WE, et al (2011) Prevention of infection by a granulocyte-macrophage colony-stimulating factor co-expressing DNA/modified vaccinia Ankara simian immunodeficiency virus vaccine. J Infect Dis 204: 164–173. 10.1093/infdis/jir199 21628671 PMC3143670

[52] Follador I, Araujo C, Orge G, Cheng LH, Carvalho LP, Bacellar O, et al (2002) Immune responses to an inactive vaccine against American cutaneous leishmaniasis together with granulocyte-macrophage colony-stimulating factor. Vaccine 20: 1365–1368. 10.1016/S0264-410X(01)00469-8 11818154

[53] Murphy ML, Wille U, Villegas EN, Hunter CA, Farrell JP (2001) IL-10 mediates susceptibility to Leishmania donovani infection. Eur J Immunol 31: 2848–2856. 10.1002/1521-4141(2001010)31:10<2848::AID-IMMU2848>3.0.CO;2-T 11592059

[54] Awasthi A, Kumar MR, Saha B (2004) Immune response to Leishmania infecction. Indian J Med Res 119: 238–258. 15243162

[55] Murray HW, Lu CM, Mauze S, Freeman S, Moreira AL, Kaplan G, et al (2002) Interleukin-10 (IL-10) in experimental visceral leishmaniasis and IL-10 receptor blockade as immunotherapy. Infect Immun 70: 6284–6293. 10.1128/IAI.70.11.6284-6293.2002 12379707 PMC130311

[56] Pereira BAS, Alves CR (2008) Immunological characteristics of experimental murine infection with Leishmania (Leishmania) amazonensis. Vet Parasitol 158: 239–255. 10.1016/j.vetpar.2008.09.015 18922635

[57] Balaraman S, Tewary P, Singh VK, Madhubala R (2004) Leishmania donovani induces interferon regulatory factor in murine macrophages: a host defense response. Biochem Biophys Res Commun 317: 639–647. 10.1016/j.bbrc.2004.03.097 15063806

[58] Carrión J, Folgueira C, Soto M, Fresno M, Requena JM (2011) Leishmania infantum HSP70-II null mutant as candidate vaccine against leishmaniasis: a preliminary evaluation. Parasit Vectors 4: 150 Available: http://www.parasitesandvectors.com/content/4/1/150. 10.1186/1756-3305-4-150 21794145 PMC3199857

[59] Soudi S, Hosseini AZ, Hashemi SM (2011) Co-administration of rectal BCG and autoclaved Leishmania major induce protection in susceptible BALB/c mice. Parasite Immunol 33: 561–571. 10.1111/j.1365-3024.2011.01318.x 21781137

[60] Courtenay O, Quinnell RJ, Garcez LM, Shaw JJ, Dye C (2002) Infectiousness in a cohort of brazilian dogs: why culling fails to control visceral leishmaniasis in areas of high transmission. J Infect Dis 186: 1314–1320. 10.1086/344312 12402201

[61] Reis AB, Teixeira-Carvalho A, Vale AM, Marques MJ, Giunchetti RC, Mayrink W, et al (2006) Isotype patterns of immunoglobulins: hallmarks for clinical status and tissue parasite density in brazilian dogs naturally infected by Leishmania (Leishmania) chagasi. Vet Immunol Immunopathol 112: 102–116. 10.1016/j.vetimm.2006.02.001 16621021

[62] Palatnik-de-Sousa CB, Barbosa AF, Oliveira SM, Nico D, Bernardo RR, Santos WR, et al (2008) FML vaccine against canine visceral leishmaniasis: from second-generation to synthetic vaccine. Expert Rev Vaccines 7: 833–851. 10.1586/14760584.7.6.833 18665780

[63] Strauss-Ayali D, Jaffe CL, Burshtain O, Gonen L, Baneth G (2004) Polymerase chain reaction using noninvasively obtained samples, for the detection of Leishmania infantum DNA in dogs. J Infect Dis 189: 1729–1733. 10.1086/383281 15116312

[64] Mettler M, Grimm F, Capelli G, Camp H (2005) Evaluation of enzyme-linked immunosorbent assays, an immunofluorescent-antibody test, and two rapid tests and gel tests for serological diagnosis of symptomatic and asymptomatic Leishmania infections in dogs. J Clin Microbiol 43: 5515–5519. 16272479 10.1128/JCM.43.11.5515-5519.2005 PMC1287801

[65] Figueiredo FB, Madeira MF, Nascimento LD, Abrantes TR, Mouta-Confort E, Passos SR, et al (2010) Canine visceral leishmaniasis: study of methods for the detection of IgG in serum and eluate samples. Rev Inst Med Trop Sao Paulo 52: 193–196. 10.1590/S0036-46652010000400005 21748226

[66] Tavares CAP, Fernandes AP, Melo MN (2003) Molecular diagnosis of leishmaniasis. Expert Rev Mol Diagn 3: 657–667. 10.1586/14737159.3.5.657 14510185

[67] Mutiso JM, Macharia JC, Kiio MN, Ichagichu JM, Rikoi H, Gicheru MM (2013) Development of Leishmania vaccines: predicting the future from past and present experience. J Biomed Res 27: 85–102. 10.7555/JBR.27.20120064 23554800 PMC3602867

[68] Beaumier CM, Gillespie PM, Hotez PJ, Bottazzi ME (2013) New vaccines for neglected parasitic diseases and dengue. Transl Res 162: 144–155. 10.1016/j.trsl.2013.03.006 23578479

[69] Cerpa-Cruz S, Paredes-Casillas P, Landeros Navarro E, Bernard-Medina AG, Martínez-Bonilla G, Gutiérrez-Ureña S (2013) Adverse events following immunization with vaccines containing adjuvants. Immunol Res 56: 299–303. 10.1007/s12026-013-8400-4 23576057

[70] Jesus-Pereira NC, Régis WCB, Costa LE, de Oliveira JS, Silva AG, Martins VT, et al (2015) Evaluation of adjuvant activity of fractions derived from Agaricus blazei, when in association with the recombinant LiHyp1 protein, to protect against visceral leishmaniasis. Exp Parasitol 153: 180–190. 10.1016/j.exppara.2015.03.027 25845753

