# Correction: Antigenicity, Immunogenicity and Protective Efficacy of Three Proteins Expressed in the Promastigote and Amastigote Stages of *Leishmania infantum* against Visceral Leishmaniasis

**DOI:** 10.1371/journal.pone.0141496

**Published:** 2015-10-20

**Authors:** Vivian Tamietti Martins, Miguel Angel Chávez-Fumagalli, Daniela Pagliara Lage, Mariana Costa Duarte, Esther Garde, Lourena Emanuele Costa, Viviane Grazielle da Silva, Jamil Silvano Oliveira, Danielle Ferreira de Magalhães-Soares, Santuza Maria Ribeiro Teixeira, Ana Paula Fernandes, Manuel Soto, Carlos Alberto Pereira Tavares, Eduardo Antonio Ferraz Coelho

The seventh author’s name is spelled incorrectly. The correct name is: Viviane Grazielle da Silva.
